# Sequencing strategies and characterization of 721 vervet monkey genomes for future genetic analyses of medically relevant traits

**DOI:** 10.1186/s12915-015-0152-2

**Published:** 2015-06-20

**Authors:** Yu S. Huang, Vasily Ramensky, Susan K. Service, Anna J. Jasinska, Yoon Jung, Oi-Wa Choi, Rita M. Cantor, Nikoleta Juretic, Jessica Wasserscheid, Jay R. Kaplan, Matthew J. Jorgensen, Thomas D. Dyer, Ken Dewar, John Blangero, Richard K. Wilson, Wesley Warren, George M. Weinstock, Nelson B. Freimer

**Affiliations:** Center for Neurobehavioral Genetics, University of California Los Angeles, Los Angeles, CA 90095 USA; Institute of Bioorganic Chemistry, Polish Academy of Sciences, Poznan, Poland; Department of Human Genetics, University of California, Los Angeles, CA 90095 USA; Department of Human Genetics, McGill University, Montreal, Canada; Department of Pathology, Section on Comparative Medicine, Wake Forest School of Medicine, Medical Center Boulevard, Winston-Salem, NC 27157-1040 USA; South Texas Diabetes and Obesity Institute, UTHSCSA/UTRGV, Brownsville, TX USA; The Genome Institute, Washington University School of Medicine, Genome Sequencing Center, St. Louis, MO 63108 USA; The Jackson Laboratory for Genomic Medicine, Farmington, CT 06001 USA; Present address: 5200 Illumina Way, San Diego, CA 92122 USA

**Keywords:** Vervet, Non-human primate, Whole genome sequencing, SNP, Linkage, Association

## Abstract

**Background:**

We report here the first genome-wide high-resolution polymorphism resource for non-human primate (NHP) association and linkage studies, constructed for the Caribbean-origin vervet monkey, or African green monkey (*Chlorocebus aethiops sabaeus*), one of the most widely used NHPs in biomedical research. We generated this resource by whole genome sequencing (WGS) of monkeys from the Vervet Research Colony (VRC), an NIH-supported research resource for which extensive phenotypic data are available.

**Results:**

We identified genome-wide single nucleotide polymorphisms (SNPs) by WGS of 721 members of an extended pedigree from the VRC. From high-depth WGS data we identified more than 4 million polymorphic unequivocal segregating sites; by pruning these SNPs based on heterozygosity, quality control filters, and the degree of linkage disequilibrium (LD) between SNPs, we constructed genome-wide panels suitable for genetic association (about 500,000 SNPs) and linkage analysis (about 150,000 SNPs). To further enhance the utility of these resources for linkage analysis, we used a further pruned subset of the linkage panel to generate multipoint identity by descent matrices.

**Conclusions:**

The genetic and phenotypic resources now available for the VRC and other Caribbean-origin vervets enable their use for genetic investigation of traits relevant to human diseases.

**Electronic supplementary material:**

The online version of this article (doi:10.1186/s12915-015-0152-2) contains supplementary material, which is available to authorized users.

## Background

Non-human primates (NHPs) are becoming increasingly valuable model species for biomedical research. NHPs share a greater degree of conservation with humans, compared to other animal models, across every level of biology from genome sequence and structure through physiology to behavior [1, 2]. At the same time, investigations of NHPs can incorporate procedures and interventions that are infeasible in human research participants, as well as control of environmental exposures, such as diet. Until very recently, however, the lack of NHP genetic and genomic resources has limited the utility of these models; in particular, progress in the field requires high-resolution genome-wide information on genetic variation comparable to that available in humans as well as large phenotyped study samples for linkage or association analyses.

As we describe here, genomic resources are now available in the vervet monkey that will enable genetic investigation of medically relevant traits, in phenotyped samples from highly abundant Caribbean-origin vervet populations. One such population is the Vervet Research Colony (VRC), an NIH-supported research resource founded in the 1970s and 1980s from 57 wild caught monkeys from the Caribbean islands of St. Kitts and Nevis [2], which has included more than 2,000 members (with DNA collected from more than 1,100 of them) in a nine-generation deep extended pedigree. We had previously reported on the construction of a genetic linkage map of the vervet using a sparse set of short tandem repeat (STR) markers [3] genotyped in a portion of the VRC pedigree. This initial genetic map enabled the provisional genetic mapping of several quantitative traits in this richly phenotyped pedigree [4–6]. SNPs identified in candidate linkage regions bolstered the evidence provided by the STRs; however, large gaps in the STR map rendered substantial portions of the vervet genome inaccessible to genetic analyses, and we lacked the high-resolution sets of polymorphisms needed to conduct genome-wide association studies (GWASs) or to systematically fine-map linkage regions to discern potentially causal variations. This insufficiency motivated the generation of the vervet reference genome assembly, constructed using a VRC monkey, which now displays a degree of sequence contiguity that, among sequenced primates, is second only to that of humans (Warren et al., submitted). The availability of the vervet reference assembly enabled the efforts described here to generate genome-wide vervet SNP sets for genetic investigations of the VRC Caribbean-origin vervet samples.

Commercially available arrays have provided an extremely inexpensive platform for genotyping genome-wide SNPs in humans and in a few model systems (such as the mouse). The insufficient market for such arrays in NHPs has necessitated the development of an alternative approach for inexpensively genotyping the vervet and other NHP models. Pasaniuc and colleagues have demonstrated that low-pass whole genome sequencing (WGS) provides a cost-effective alternative to SNP genotyping arrays for GWASs [7]. We reasoned that a similar strategy could be employed in the vervet. While Pasaniuc et al. used the extensive reference data available from human populations to determine genotypes, we planned to leverage the information available from the VRC pedigree structure to enable accurate assignment of genotypes. We describe here the use of a hierarchical sequence coverage strategy: deep sequence in 17 VRC monkeys to infer WGS-based genotype calling in more than 700 additional descendant VRC monkeys sequenced at a lower depth. We then indicate how, from these genotypes, we developed genome-wide panels of common vervet SNPs that will be useful for both linkage and association analyses of medically relevant traits.

## Results

The generation of common SNPs for linkage and association studies in the VRC started from a preliminary study, in which we systematically assessed the impact of coverage depth on genotyping accuracy depending on position in the pedigree, using sequence data from 105 monkeys. We used these data in a down-sampling experiment to inform our selection of coverage depth for sequence analysis of 620 additional monkeys. We then followed four main stages of analysis in the entire set of 725 monkeys: 1) identifying unequivocal segregating sites for common variants in 17 monkeys sequenced at high depth; 2) calling genotypes pedigree-wide at the SNPs identified in Stage 1 in all 725 monkeys; 3) performing sample-level quality control (QC) using SNPs called in Stage 2; 4) establishing SNP sets for genetic analyses by thinning the common SNPs from Stage 2, in monkeys that passed QC in Stage 3. We describe the results for each of these stages, indicating the number of SNPs that we removed, in each stage, by applying a series of QC filters.

**Fig. 1:**
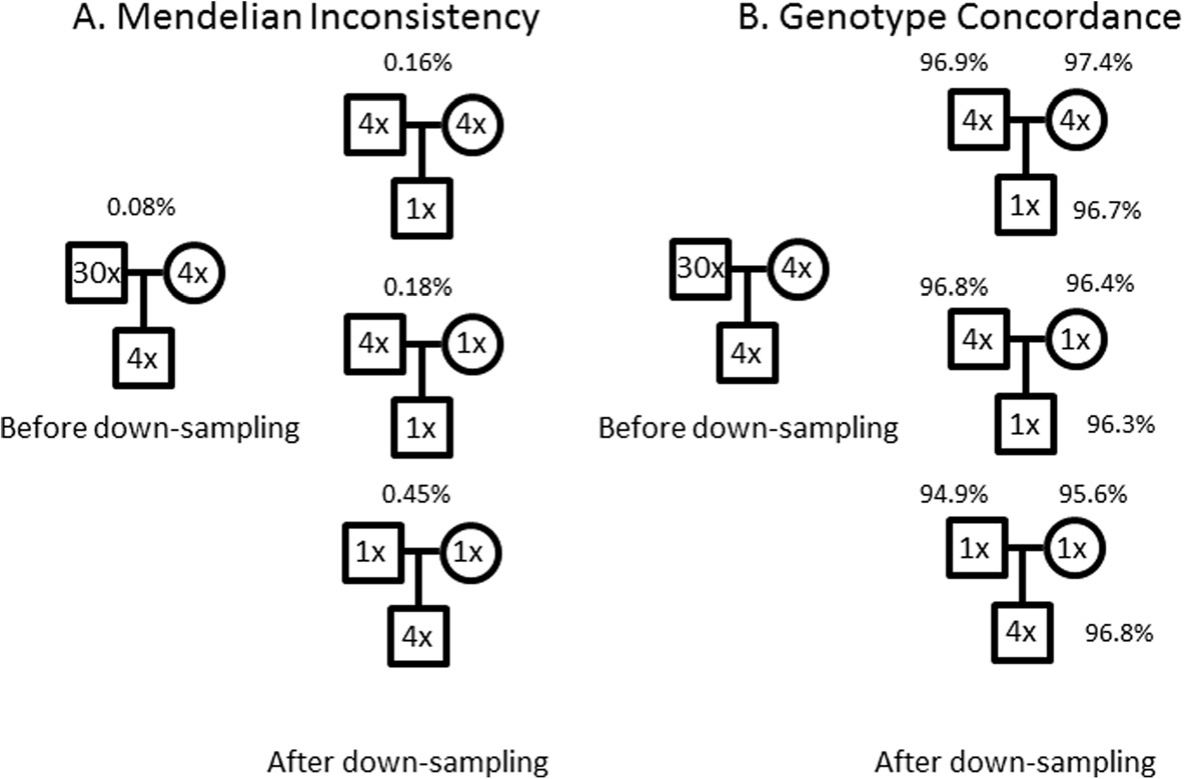
Using the variant data from the WGS of the trio shown on the left, we evaluated three different down-sampling schemes, drawn on the right, to determine a pedigree-wide strategy for selecting monkeys for medium (4X) or low (1X) sequencing coverage. **a** The frequency of Mendelian errors in a trio increases in all three down-sampling experiments compared to the original data; however, the increase in error rate is greatest when both parents are low coverage and the child is medium coverage. **b** The percentage of concordant genotype calls between original data and down-sampled data is lowest when both parents are low coverage and the child is medium coverage. The percentages shown for both the rate of Mendelian inconsistency and for genotype concordance represent averages over three down-sampling experiments

Down-sampling analysis showed that sequencing both parents at 4× (and their offspring at 1×) resulted in a much lower rate of Mendelian inconsistencies and a higher degree of genotype concordance with non down-sampled genotypes (in parents) than a strategy in which both parents are sequenced at 1X and their offspring at 4× (Fig. 1). Importantly, the intermediate strategy (sequencing one parent at 4X, one parent at 1X, and the child at 1×) has a similar Mendelian inconsistency rate and genotype concordance (for all three trio members) to the strategy in which both parents in the trio are sequenced at 4×, suggesting the possibility of achieving further gains in cost effective recovery of genotypes. We also observed, in genotype analysis of the initial 105 monkeys that lowering the sequencing coverage of any single monkey has very little impact on the accuracy of genotyping of pedigree members beyond the trios of which s/he is a member (data not shown).

Considering the results of the down-sampling analysis, we used a greedy algorithm (see Methods) to rank the pedigree members based on the number of their direct descendants included in the set of 725 monkeys that comprised the WGS sample, and employed this ranking as the primary rationale for including them in either high coverage (>25× average), medium coverage (>4× average), or low coverage (>1× average) sequencing bins. Based on this algorithm (and including the 105 monkeys sequenced in our preliminary study) we assigned 16 monkeys to high coverage, 407 to medium coverage, and 302 to low coverage WGS.

**Fig. 2:**
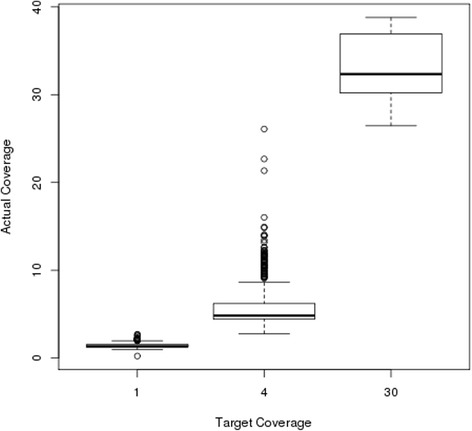
Boxplot of actual sequencing depth versus planned sequencing depth for the 725 monkeys in the WGS study

Given improvements in technology over the course of the project, our sequencing protocol resulted, for a substantial proportion of samples, in considerably deeper coverage than had been our target (summary sequencing statistics for each monkey are given in Additional file 1: Table S1). As a result, the variation in sequencing depth, across the pedigree, is more accurately represented as a continuum than as three discrete bins (Fig. 2). For example, among the 406 monkeys targeted for 4–6× sequencing we achieved >20× average depth for 3 monkeys; 10–20× depth for 61 monkeys; and 6–10× depth for 36 monkeys. We considered “high coverage” monkeys to be a set of 17 monkeys, each with >25× average coverage, including the 16 monkeys initially chosen for high-depth sequencing and an additional monkey sequenced at a higher depth than originally planned.

In Stage 1 we discovered 13,550,322 SNPs in the 17 high coverage monkeys, and removed 9,314,561 SNPs from further investigation based on different QC-associated metrics, with low polymorphism content (minor allele frequency [MAF] <25 %) in these 17 monkeys being the largest factor, leading to the removal of about 6M SNPs (Table 1). For the 4,235,761 SNPs that passed through the Stage 1 filters, we called genotypes in all 725 sequenced monkeys (Stage 2). In Stage 2 we retained all SNPs that had a MAF ≥ 10 % (as estimated in all 725 monkeys) and performed a series of QC steps (Table 2) that left us with 3,369,989 high-quality common autosomal SNPs for construction of genome-wide association and linkage panels.

**Table 1:** Filtering and QC procedures in Stage 1: identifying unequivocal segregating sites. Stage 1 started with 13,550,322 sites and after QC ended with 4,235,761 sites

QC filtering procedure	Number of variants removed
Multi-allelic or multi-nucleotide	1,110,071
Cumulative coverage outside of twofold range of global median coverage	1,158,822
MAF in 17 monkeys <25 %	6,859,481
>0 % missing data	164,781
Within 5 bp of another site	21,406
TOTAL	9,314,561

**Table 2:** Filtering and QC procedures in Stage 2: calling genotypes in all 725 monkeys at the unequivocal segregating sites identified in Stage 1. Stage 2 started with 4,235,761 sites and ended with 3,369,989 sites

QC filtering procedure	Number of variants removed
Not passing SAMtools filters (“mpileup -S -D -q 30 -Q 20”, “vcfutils.pl varFilter -w 10 -d 3 -D 12740 -e 0–2 0”)	209,826
Cumulative coverage outside of twofold range of global median coverage	20,843
MAF in 723 monkeys <10 %	10,766
Missing >50 % of data	105
Too few (<3) loci in 3Mb regions, not enough for TrioCaller to work.	1,360
Loci unmapped or not mapped uniquely during LiftOver	32,419
Filtered out by GATK’s FilterLiftedVariants	4,094
Whole contig removed for contigs with >1 chromosome switching events per 100 loci	6,208
LiftOver MapScore <0.5	61,721
Loci mapped to the same coordinate in the new reference genome	4
Alignment: identified regions of poor alignment (mapping quality <2- or coverage >2-fold range of global median depth) and masked these genotypes as missing. Sites with >50 % missing in 4X and above monkeys are removed	438,423
Sex chromosome SNPs	65,271
>=5 Mendel errors in parent–child comparisons	8,563
>60 % heterozygous calls	6,201
Total	865,772

In Stage 3 we used SNPs that passed through Stage 2 to perform sample-level QC and to refine the pedigree structure. Based on discrepancies between known pedigree relationships and identity by descent (IBD) estimated from linkage disequilibrium (LD)-pruned SNPs, and on concordance between WGS data and a set of SNP genotypes generated for another study with independent methods [5], we excluded data for three monkeys that we strongly suspected represented contamination or mislabeling of DNA samples. The information from pairwise IBD relationships (together with VRC records, see Methods) also enabled us to identify the parents for all but one of the 174 sequenced monkeys whose parentage had previously been unknown. We excluded from further analyses the single monkey for which we could not identify parents, leaving 721 monkeys for analysis in Stage 4.

In Stage 4 we then obtained final SNP sets for association and linkage analysis by thinning the final pedigree-wide common SNP set generated in Stage 2, removing variants (assessed in 50 marker windows along each autosome) with redundant genetic information (as evidenced by LD, at r^2^ thresholds appropriate to either association or linkage mapping). We conducted the steps in Stage 4 to provide the community conducting vervet research in the VRC with well-vetted sets of polymorphisms that will facilitate their performance of genetic analyses; it is not our intent to utilize these sets to construct vervet-specific genotyping arrays. By LD pruning at r^2^ < 0.9 we obtained a set of almost 500,000 SNPs suitable for genome-wide association analysis, and by LD pruning at r^2^ < 0.4 obtained a less dense set of almost 150,000 SNPs suitable for linkage analysis. We then finalized these SNP sets by removing SNPs for which pedigree-wide checks revealed Mendelian inconsistencies (554 and 159 in the 500K and 150K sets, respectively). Both SNP sets (Table 3) include predominantly highly polymorphic SNPs (mean heterozygosity of 0.45–0.47 for each of the 29 autosomes) that are densely placed across the genome (mean gap is 5.1 Kb for the 500K set and 17.5 Kb for the 150K set).

**Table 3:** Characteristics of the two mapping sets of markers derived in Stage 4

	Approx. 500K mapping set					Approx. 150K mapping set				
CHR	N	SNP/Mb^a^	Max Gap^b^ (BP)	Mean Gap^b^ (BP)	Mean R2^c^	N	SNP/Mb^a^	Max Gap^b^ (BP)	Mean Gap^b^ (BP)	Mean R2^c^
1	25863	205.2	1074224	4873	0.38	7695	61.1	1112341	16380	0.31
2	16774	185.7	3730217	5385	0.40	4403	48.8	3731818	20515	0.34
3	18390	199.7	1144078	5007	0.38	5653	61.4	1176302	16291	0.33
4	15560	209.3	1663269	4777	0.39	5476	73.7	1689958	13576	0.34
5	15940	211.4	1150416	4730	0.38	4674	62.0	1275087	16132	0.32
6	10112	198.7	1020214	5032	0.36	2792	54.9	1030612	18227	0.29
7	24288	178.9	1518445	5590	0.40	7026	51.8	1631219	19310	0.35
8	28272	220.8	2004329	4530	0.37	9192	71.8	2598031	13933	0.33
9	21550	171.6	1066472	5828	0.38	6456	51.4	1089666	19453	0.33
10	20112	156.4	1043042	6393	0.39	6104	47.5	1050307	21054	0.37
11	25512	198.5	2199143	5038	0.39	7530	58.6	2424911	17072	0.32
12	17587	162.0	1420166	6171	0.39	5615	51.7	1520086	19331	0.34
13	18794	191.1	1516380	5234	0.38	5934	60.4	1518768	16573	0.33
14	19105	177.6	1817866	5632	0.44	5943	55.3	1830044	18099	0.39
15	18395	200.5	1542801	4988	0.39	5110	55.7	1545377	17952	0.32
16	14040	186.9	1023476	5350	0.36	3698	49.3	1026661	20304	0.30
17	15640	217.3	1093222	4602	0.39	4292	59.7	1122085	16754	0.34
18	15291	211.6	1330661	4726	0.41	4623	64.0	1542198	15620	0.35
19	5596	173.5	476395	5765	0.41	1416	43.9	554050	22793	0.35
20	27113	209.3	459070	4778	0.35	8034	62.1	711003	16094	0.28
21	18878	149.6	1477644	6685	0.41	6175	48.9	1502779	20435	0.37
22	15859	158.4	258174	6314	0.43	5064	50.6	422458	19777	0.40
23	16564	202.4	391245	4940	0.38	5118	62.6	732327	15985	0.32
24	18746	223.4	225900	4476	0.37	4889	58.3	325648	17162	0.32
25	18731	221.2	238967	4522	0.37	5563	65.7	421038	15228	0.30
26	12435	217.8	215585	4593	0.38	3324	58.3	270040	17166	0.31
27	12055	259.1	284037	3860	0.37	3470	74.8	425185	13379	0.31
28	4122	207.4	199874	4822	0.43	1046	52.7	379794	19003	0.37
29	5839	252.6	112574	3960	0.35	1652	71.5	211468	13998	0.29

^a^Number of SNPs per Mb

^b^Max and mean distance between consecutive SNPs, in basepairs

^c^Average of pairwise estimates of windows of five markers

To further facilitate linkage analyses of the VRC pedigree we used 9,752 SNPs, LD-pruned from the 150K SNP set, to construct multipoint identity by descent (MIBD) files. We estimated a genetic map for these SNPs by interpolating from the 338 autosomal STRs (Additional file 2: Figure S1) that constituted the vervet STR genetic map [3], deriving the physical position of these STRs from the vervet reference assembly. We then estimated the probability of IBD between all pairs of vervets, including monkeys without sequence data (estimated using pedigree connections), at 1-cM intervals through the genome, for a total of 2,899 estimates for each pair of monkeys. The MIBD estimates are available upon request.

## Discussion

The completion of the WGS of 721 VRC monkeys and the generation of the SNP sets reported here will enable genome-wide genetic analyses of numerous quantitative traits already assessed in this pedigreed colony.

The development of this sequencing-based resource will almost certainly be followed in the very near future by similar mapping tools in large pedigrees in other NHP species, including rhesus macaques [8] and baboons [9]. As many of the traits assayed in the VRC are also available in these pedigrees, high-resolution comparative analyses to elucidate genotype-phenotype relationships may soon be feasible.

Unlike the pedigrees sequenced in other NHP species, the VRC derives from a clearly demarcated and homogeneous ancestral population, that of the more than 50,000 feral vervets resident on St. Kitts and Nevis [2]. Because the monkeys used to found the VRC were trapped from numerous sites across these small islands, it is hypothesized that the VRC variant catalogue incorporates most of the genetic variation represented in the island populations. This expectation is particularly applicable to the SNP sets, given the initial prerequisite that SNPs included in these sets displayed a MAF of > 25 % in the 17 high coverage monkeys. As DNA samples, biomaterials, and phenotype data are already available for nearly 800 island monkeys, and collections there are ongoing, it may soon be possible to conduct GWASs or fine-mapping association studies in these population samples by analyzing the SNPs identified in the VRC [2, 5]. Because the samples available from the island vervet populations are more independent from one another than the VRC samples sequenced to date, it will be possible to more accurately delineate blocks of LD, genome wide. This delineation will enable the modification of the current 500K association panel, to ensure that future GWASs adequately tag these blocks.

Further analyses of the already obtained VRC WGS data will add in other important ways to the genetic analysis resources described here. First, we anticipate that the vervet genome assembly will undergo further refinement; as a result, we will obtain a more complete delineation of vervet structural genomic variation and also close current gaps in SNP coverage. Second, we will soon be able to use the vervet gene annotation to predict the functional impact of coding variants, for example, to identify missense variants predicted to have a deleterious effect. Given the extensive inbreeding loops within the 2,000-monkey VRC pedigree and its rapid expansion from 57 founders, we hypothesize that many such variants that may have been carried by only a single founder could now be relatively frequent among the 721 sequenced VRC monkeys. Such variants could be the starting point for “phenome screening” of the VRC to identify traits on which they may have a substantial impact [10]. To conduct such studies we will need a high degree of confidence in the calling of such variants and the assignment of genotypes, particularly in those monkeys sequenced at intermediate and low depths; to obtain such confidence, we will re-sequence several hundred VRC monkeys using an independent method (targeted capture of multiple genome regions) and then evaluate concordance with the current genotypes. Finally, we predict that this resource will enable the extension to vervet of evolutionary analyses that, among primates, have to date mainly been limited to humans [11]. By quantifying, in the vervet genome, segregating mutation rates for different classes of variants (for example, those that are protein coding, regulatory, or repetitive), we may obtain a clearer picture of similarities and differences between the evolutionary histories of humans and NHPs.

## Conclusions

We have demonstrated the effectiveness of a strategy that combines high, medium, and low coverage WGS for generating the first high-resolution genome-wide SNP resource for an NHP species. This resource will enable linkage and association studies that take advantage of the rich phenotype data available for large samples of Caribbean-origin vervet monkeys.

## Methods

### Overview of WGS strategy and methods

We selected, in two phases, 725 monkeys for WGS from a total of 1,138 VRC monkeys with DNA available in the Biological Sample Repository at UCLA. In the preliminary study phase we selected 105 monkeys for initial WGS, to evaluate the relationships among sequencing depth, variant identification, Mendelian error rate, and pedigree structure. The monkeys selected for the preliminary study included i) four monkeys for high coverage (about 30×) WGS from the surviving generation that was closest to the VRC founder generation, all having multiple (14–115) descendants available for WGS; and ii) 101 monkeys for medium coverage (about 4–6×) WGS from “middle” generations, emphasizing either those with multiple descendants available for WGS or those with extensive biological samples available for phenotypic investigations.

We then selected for sequencing in the next phase of the project an additional 620 monkeys, chosen based either on i) their having multiple descendants or ii) their having been assessed for ≥ 7 phenotypes or having multiple biological samples available. Results from analysis of the preliminary study enabled us to devise an algorithm to rank these 620 monkeys for assignment to high coverage, medium coverage, and low coverage (about 1×) WGS, where monkeys with a higher rank would be sequenced at greater depth than monkeys with a lower rank.

We identified segregating sites in 17 high coverage (30×) monkeys and then called genotypes at these sites in the full set of 725 monkeys (105 + 620). We then conducted a series of filtering steps to ensure that we were retaining high-quality, polymorphic sites, including genotype calling that incorporated linkage disequilibrium (LD) and Mendelian constraints. By thinning these filtered high-quality sites to remove markers with redundant genetic information (based on LD), we generated the final association and linkage SNP sets. The following sections provide details of all steps.

### Generation of WGS data

We generated the WGS data for 725 monkeys from genomic DNA provided by the Systems Biology Sample Repository at UCLA. We have generated 100 base pair short read sequences on the Illumina HiSeq2000 instrument from short insert libraries (200–400 bp) for 725 vervets at The Genome Institute (TGI) at Washington University in St. Louis and the McGill University and Genome Quebec Innovation Centre. We have submitted all WGS sequences to the Sequence Read Archive (SRA) under the NCBI BioProject number PRJNA240242.

### Preliminary study

#### Read mapping of preliminary sequence data and refining genotype calls

We followed the published protocol of the 1000 Genomes Project [12] for read mapping of the 105 WGS produced in the preliminary study, with minor modifications related to read trimming detailed in Additional file 3: Supplementary methods Initial variant calling of the alignment files used SAMtools [13]. Due to the size and complexity of the VRC pedigree, we were unable to refine these genotype calls using pedigree-aware methods such as Polymutt [14]. We therefore broke the pedigree into units (trios and parent-offspring duos) that could be analyzed with the programs TrioCaller [15] and Beagle 4.0 [16]. We broke full sibships into distinct trios by replicating parental information. Similarly, for male monkeys who have had offspring with multiple different female monkeys, we replicated this information for each offspring.

With the pedigree broken into trios, duos, and singletons, we used Beagle to first obtain a pedigree-specific haplotype reference panel from the distantly related high coverage monkeys. We then used this reference panel to phase and impute genotypes of all sequenced members of the pedigree. Finally, we used TrioCaller to refine previous haplotypes by incorporating trio constraints and read depth information.

After this step we derived two consensus haplotypes for each monkey, splitting replicated haplotypes from the same monkey into two clusters, corresponding to two chromosomes, based on the Hamming distance between haplotypes. Within each cluster we built a consensus haplotype by accepting the majority call at each locus. We applied the above procedure only to SNPs on autosomes, as the Mendelian transmission model of Beagle and TrioCaller is not applicable to chromosomes X and Y.

#### Down-sampling analysis to determine coverage strategy

We used the preliminary data to evaluate the impact of WGS coverage in monkeys, in relation to their position within the pedigree, by conducting analyses of down-sampling (reducing the coverage of a monkey, *in silico*, to a prespecified level). We considered parent-offspring units for this analysis and determined the relative importance of selected parents, compared to their offspring, in influencing the accuracy of genotype assignments. We then extrapolated the results to the entire pedigree.

The down-sampling analysis included three *in silico* experiments, each repeated three times, on preliminary WGS data. All three down-sampling experiments involved reducing the coverage of one trio with the best available coverage (father sequenced at 30×, mother at 4–6×, offspring at 4–6×) to three different settings. To down-sample the level of coverage of one monkey *in silico* to the prespecified target coverage, we uniformly sampled a fraction (target-coverage/existing-coverage) of all its existing sequencing reads that had been aligned to a reference genome; this step generated a new alignment file with reduced coverage. We left the alignment files of other monkeys in the pedigree unchanged, and then applied the same calling procedure as that described above to this revised set of 105 alignment files. We then calculated and compared the Mendelian error rates and genotype concordance rates before and after down-sampling.

#### Sequence coverage ranking

The down-sampling experiment enabled us to evaluate the effect of sequencing coverage on SNP Mendelian error rates in the context of a pedigree, and results suggested that a ranking strategy, based on the number of a monkey’s direct descendants, would be useful to determine sequence coverage of the remaining 620 monkeys. The rank of a monkey would reflect its importance in holding down the error rate of SNPs of all sequenced monkeys, not just the error rate of the monkey with its offspring/parents. We devised a greedy algorithm to rank monkeys in terms of the impact that their sequencing would have on the frequency of errors in SNP calling, over the entire pedigree. We applied this algorithm to all 725 monkeys, assigning the 620 monkeys not yet sequenced to different sequencing coverage classes based on the ranking. This procedure involved the following steps:

1. Construct the pedigree as a directed graph. Each node represents a monkey. Each edge goes from one parent (father/mother) to a child. Remove monkeys that will not be sequenced.
2. Initialize an empty set (child-set). It will store the ID of monkeys for whom one parent has been already ranked. No identical IDs are stored in it.
3. Start with the monkey that has the most direct descendants. Assign that monkey “rank 1” and add its direct descendants into the offspring-set.
4. Select as “rank 2” the remaining monkey that would most increase the size of the offspring-set; if there are ties (contribution to the offspring-set is identical for >1 monkey), older monkeys are given lower rank.
5. Stop when there are no monkeys left to be ranked.

### Execution of the sequencing strategy

#### Sequence alignment of combined data

We conducted read mapping, as described above, of the entire set of 725 WGS monkeys; this step included re-mapping of the 105 monkeys from the preliminary study, described above, given updates to the reference genome assembly since we had produced the data from that study. We performed read mapping for the entire set using the final pre-submission version of the Vervet Reference Genome, consisting of 2,199 scaffolds, and followed the procedures described above for read trimming, alignment, and marking of duplicates.

Additionally, we then performed a base quality score recalibration, and local indel realignment procedures [17]. Because a set of known variants is not available in vervets, we generated an initial “bootstrap” set of SNP and indel variants by running SAMtools on 82 monkeys with coverage >=10× and filtering out all loci outside of a twofold median-depth range or missing fraction more than 50 %. We then input the positions of these loci to GATK’s Base Quality Score Recalibration (BQSR) and local-indel realignment procedures [17].

#### Stage 1: Identify unequivocal segregating sites from 17 high coverage monkeys

To assemble SNP sets for linkage and association analyses, we wished to include only highly polymorphic and reliable variants. We therefore focused on identifying such polymorphic variants among the most deeply sequenced monkeys. We used a local-assembly-based haplotype caller, Platypus [18], to discover variants from alignment files of 17 high coverage (HC) monkeys. This caller, unlike single-site callers (SAMtools and GATK’s UnifiedGenotyper) used in the 1000 Genomes Project, employs two measures to reduce genotyping errors: 1) It assembles reads within a window (set to 500 Kb in our case) into two haplotypes and then discovers variants by comparing the assembled haplotypes and the reference genome; 2) by local haplotype assembly, it corrects the indel-induced local wrong alignment and excludes wrongly placed reads from the assembled haplotypes. We applied a series of filters (described in Additional file 3: Supplementary methods) to the sites identified in the 17 HC monkeys to reduce the set of variants passed on to Stage 2. Importantly, to retain polymorphic markers that would be the most useful in genetic mapping, we filtered out variants with minor allele frequency (MAF) < 25 % in the set of 17 HC monkeys. As we selected these 17 HC monkeys primarily because they are ancestral to a substantial proportion of the current pedigree, we considered it likely that variants observed in only one or a few monkeys in this set would not be well represented in the pedigree overall. We therefore implemented this relatively high MAF filter to maximize the identification of variants that would be common throughout the pedigree and therefore most useful for genome-wide linkage or association analyses.

#### Stage 2: Assign genotypes at the SNPs to all monkeys, using SAMtools, with refinement using TrioCaller and Beagle

We used SAMtools to assign genotypes to all 725 sequenced monkeys, at the SNPs identified in Stage 1. As described for the preliminary study, we subsequently refined SAMtools calls with methods that used LD and Mendelian constraints (TrioCaller and Beagle). We then discarded loci whose coverage is outside the twofold range of global median coverage, loci whose minor allele frequency is below 10 % (as estimated in the full set of 725 monkeys), and loci with >=50 % missing calls.

We lifted over the scaffold-based coordinates of VRC SNPs retained in Stage 2, from the pre-submission version of the reference assembly, to the chromosome coordinates of the NCBI-released version (Chlorocebus_sabaeus 1.1, GCA_000409795.2), using GATK’s LifeOverVariants procedure. We applied additional filters to remove SNPs with questionable positions (details are given in Additional file 3: Supplementary methods).

#### Stage 3: Sample-level QC of WGS data and refinement of pedigree structure

Once we had obtained genotypes for all 725 monkeys at the variant sites identified in the 17 high coverage monkeys, we performed sample-level QC to identify possible sample mix-ups or contamination. Using PLINK [19], we LD-pruned the SNPs that passed Stage 2, employing a very low LD threshold (pairwise r^2^<=0.1, window size=50, shift=20). We then estimated the pairwise IBD sharing for all pairs of monkeys using this set of roughly independent SNPs. We then compared the PLINK IBD estimates with the kinship estimates that were entirely based on the pedigree structure, as estimated by SOLAR [20], and identified those monkeys frequently involved in discordant pairs. We also checked the genotype concordance between the WGS SNP set and a dataset of SNP genotypes, generated previously, using independent methods, for fine mapping of QTL in the VRC [4]; for this comparison we used 415 monkeys for which we had genotypes from both the WGS SNPs and the previously generated SNPs.

At the start of the WGS studies, parentage was known for monkeys born prior to 2008; this information derived from microsatellite-based genotyping [3] together with observational data. For monkeys born during or after 2008 (N=174) we attempted to identify parentage using the pairwise IBD relationships (estimated with WGS data as described above) between these monkeys and all others.

To assign parentage we required: 1) identification of levels of IBD sharing expected between parent-offspring, with at most one monkey of each gender demonstrating such a level of sharing to monkeys of unknown parentage; 2) for mothers, concordance between the WGS IBD information with data from colony records on observed mother-infant behavior; 3) for fathers, concordance between WGS IBD information and data from colony records indicating a set of possible fathers (based on their sharing housing with the mothers and infants in question); 4) a plausible age difference between putative parents and offspring (within a range of 4–15 years).

#### Stage 4: Generating the final SNP mapping sets and multipoint IBD files

After removing data from samples suspected of contamination or mislabeling, and updating the pedigree based on newly identified parent-offspring relationships, we repeated the genotype call refining (Beagle + TrioCaller) step, described in Stage 2. We then applied the following filters to generate our final mapping sets: 1) removed SNPs with >=5 Mendel errors between parents and offspring; 2) removed SNPs with excess heterozygosity (heterozygous fraction>0.6); 3) marked the remaining (sporadic) parent-offspring Mendelian-error genotypes as missing; 4) removed SNPs with missing rate >5 %.

We then thinned the SNP dataset to generate two genetic mapping marker sets, one appropriate for association (retaining SNPs with pairwise r^2^<=0.9) and one appropriate for linkage mapping (retaining SNPs with pairwise r^2^<=0.4). In conducting the pruning for both marker sets we used PLINK, with window size=50 and shift size =20. Lastly we used PedCheck [21] to detect SNPs that showed pedigree-wide Mendel errors and removed them from both mapping sets. We deposited in NCBI the WGS-based genotype data from 721 VRC vervets (used to construct the 500K and 150K SNP mapping sets); these data are publically available under BioProject PRJEB7923, and browsable at [22].

To further enhance the utility of the SNP sets, we used LOKI [23] to construct multipoint identity by descent (MIBD) files. These files summarize the probability that, at a particular location in the vervet genome, a pair of sequenced monkeys share genotypes IBD. These estimates are the basis for multipoint variance component linkage analysis in SOLAR, the most widely used approach for pedigree-based QTL analysis. In order to evaluate MIBD, which is very computationally intensive, we used LD pruning to obtain a subset of SNPs from the linkage mapping SNP set. This reduced set of markers was adequate for MIBD evaluation, as the close connections among the inbred pedigree has led to IBD sharing over long chromosomal segments.

## Additional files

Additional file 1: Table S1. Presents target read depth and actual read depth for sequenced animals. Additional file 2: Figure S1. Presents the number of SNPs in intervals between STR markers from the initial vervet genetic map. Additional file 3:
**Supplementary methods.**
