# Supplementary material for: Sequencing strategies and characterization of 721 vervet monkey genomes for future genetic analyses of medically relevant traits
Source: BMC Biol. 2015 Jun 20;13:41. doi: 10.1186/s12915-015-0152-2 (PMC4494155; doi:10.1186/s12915-015-0152-2)
Supplement: Additional file 2: Figure S1. — Presents the number of SNPs in intervals between STR markers from the initial vervet genetic map. [file 12915_2015_152_MOESM2_ESM.pdf]

Figure S1. Number of SNPs in inter-STR intervals. Locations of STRs on the vervet genetic map (cM) are indicated by dashed vertical lines. Midway between each adjacent STR is plotted the number of SNPs in that interval in the 500K mapping set (black) and the 150K mapping set (red).

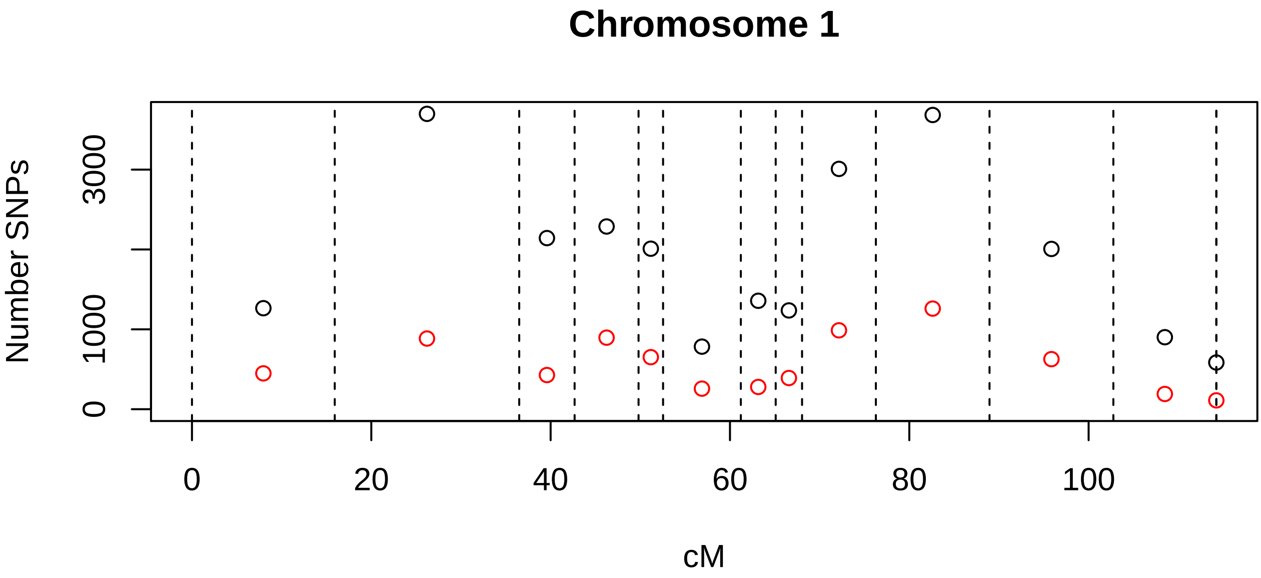
Chromosome 1
Number SNPs
0
1000
3000
0
20
40
60
80
100
cM

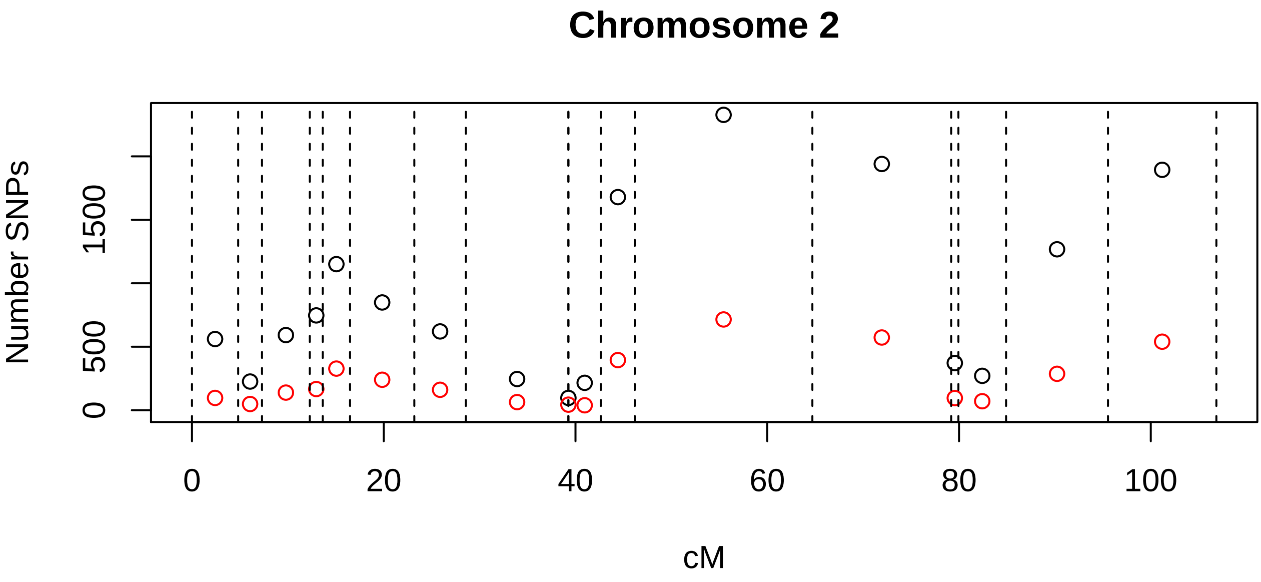
Chromosome 2
Number SNPs
0
500
1500
0
20
40
60
80
100
cM

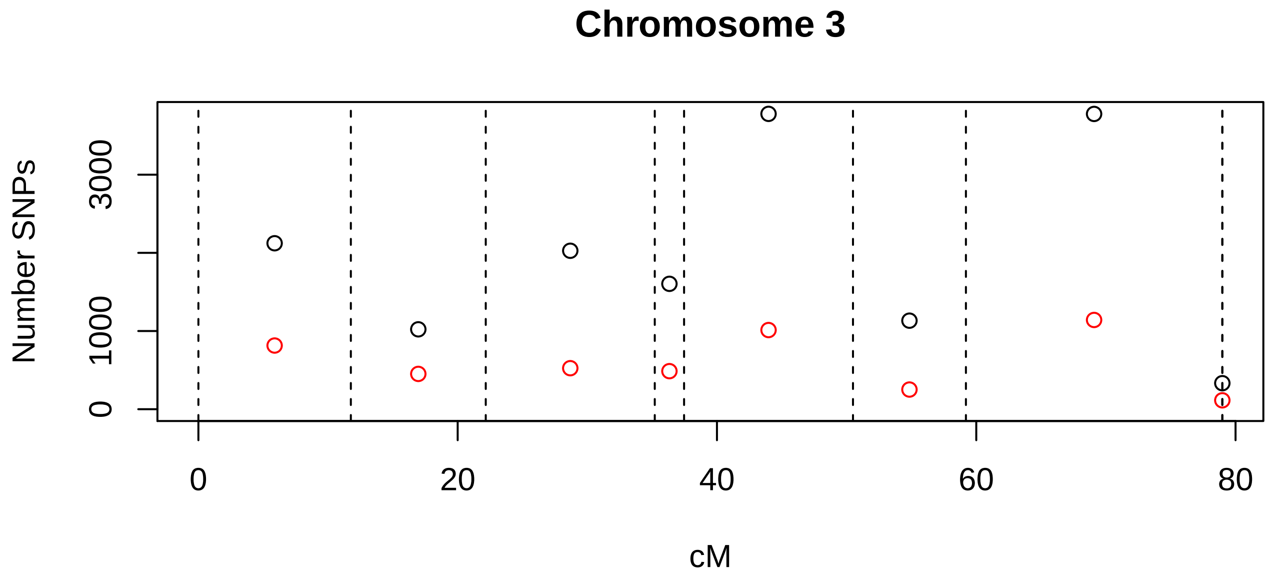
Chromosome 3
Number SNPs
0
1000
3000
0
20
40
60
80
cM

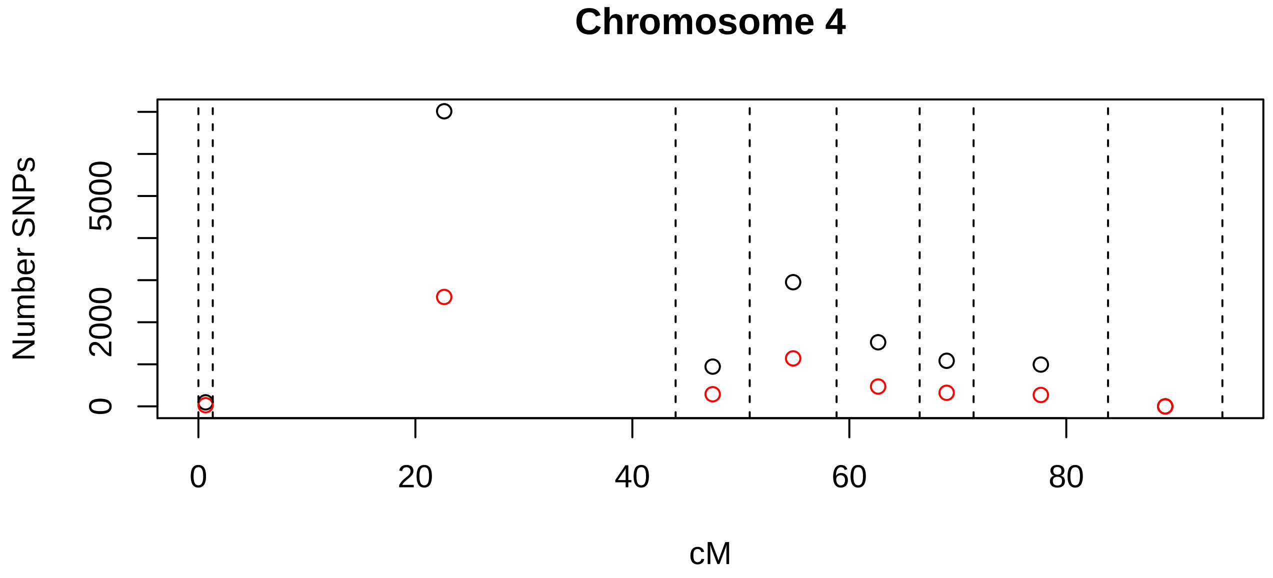
Chromosome 4
Number SNPs
0
2000
5000
0
20
40
60
80
cM

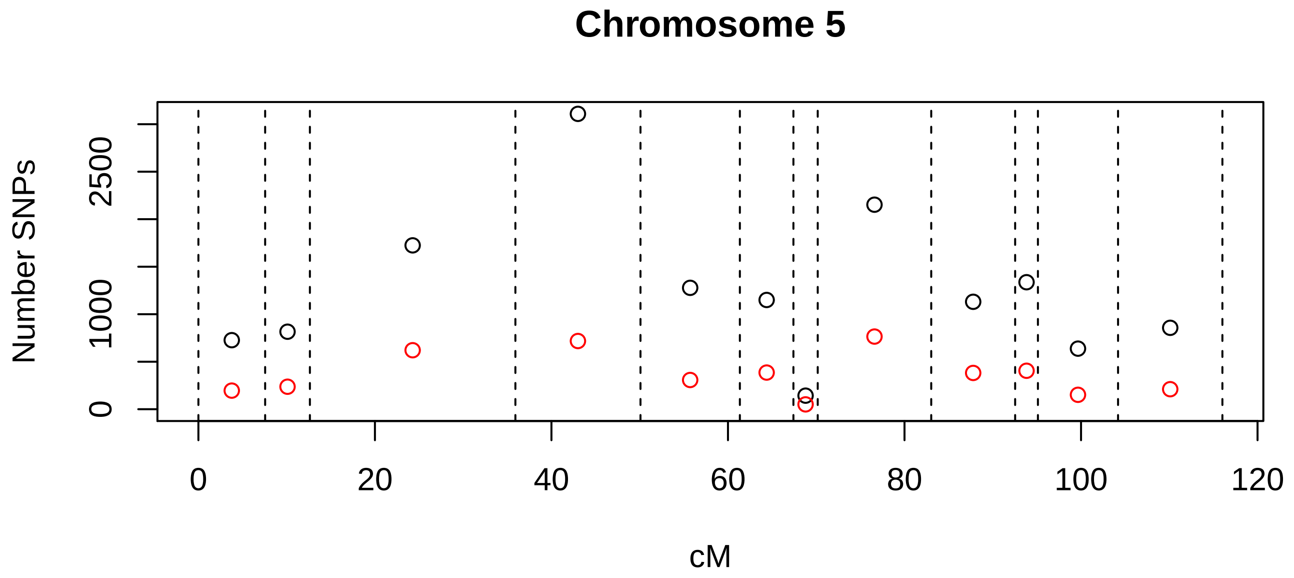
Chromosome 5
Number SNPs
0
1000
2500
0
20
40
60
80
100
120
cM

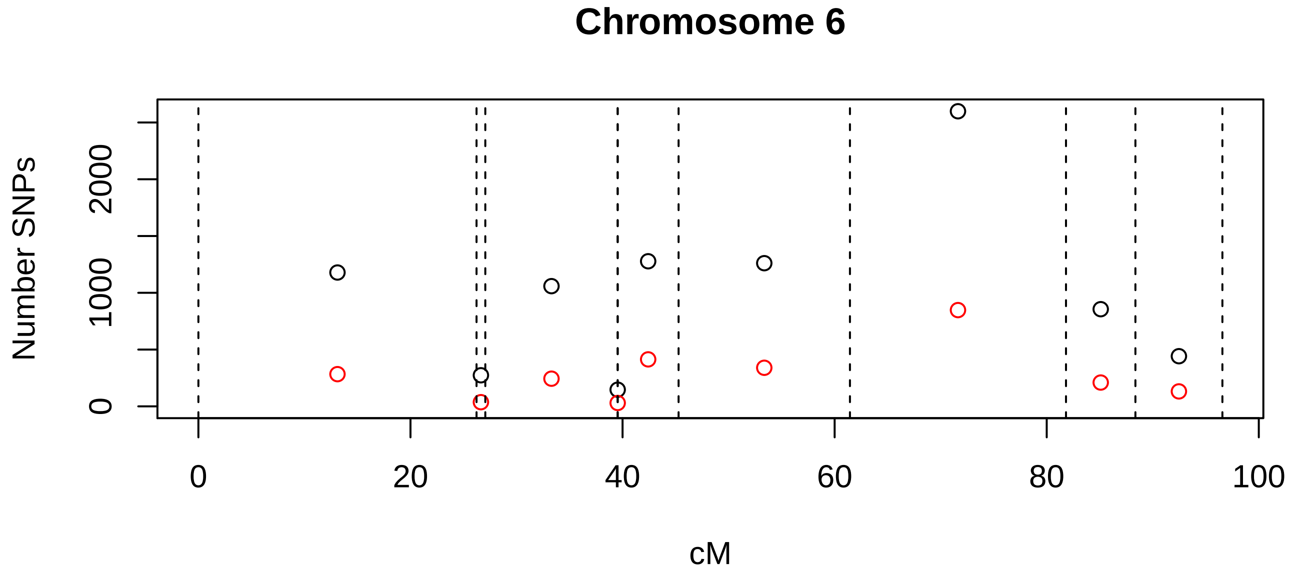
Chromosome 6
Number SNPs
0
1000
2000
0
20
40
60
80
100
cM

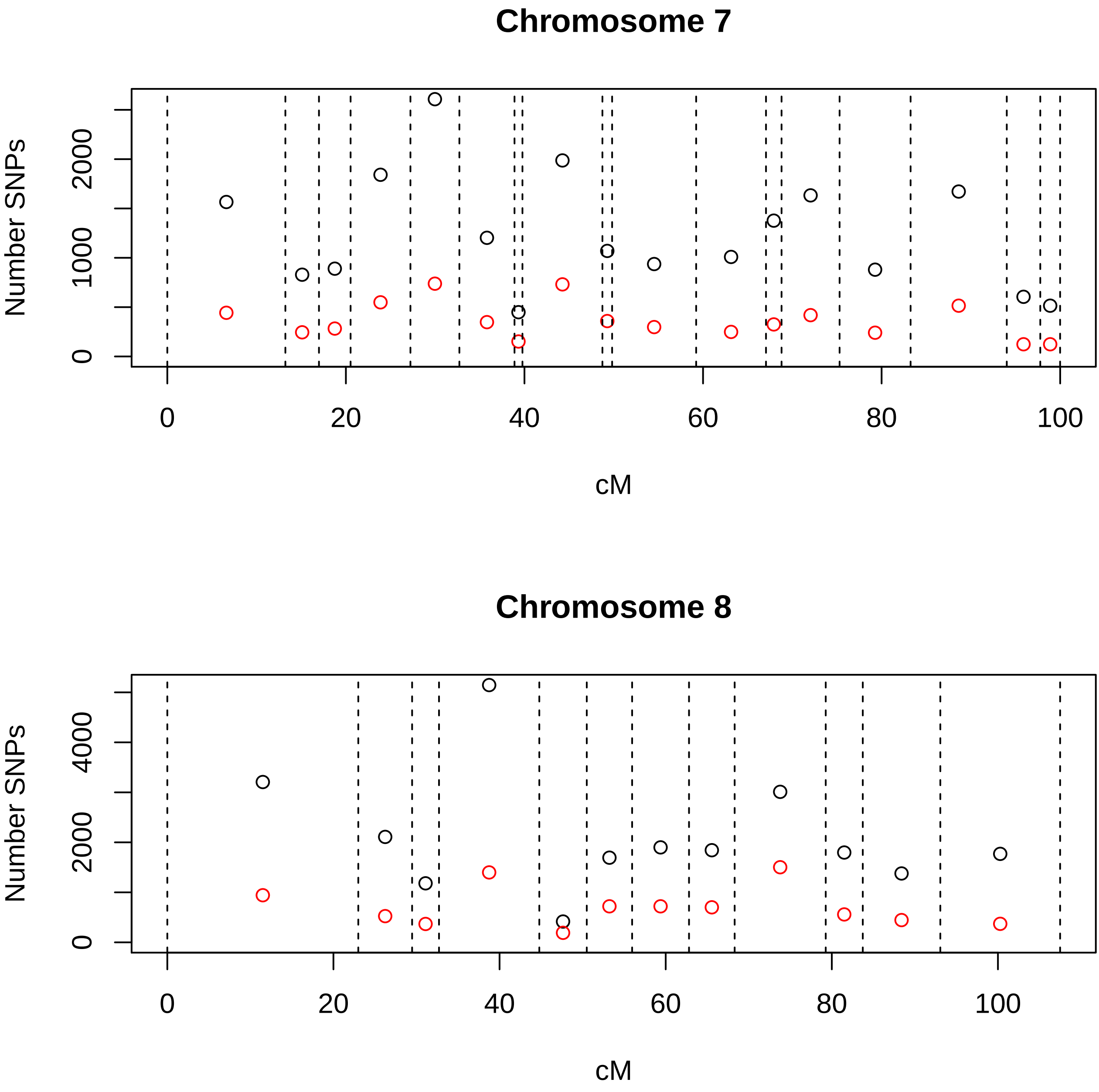

Chromosome 7
Number SNPs
0
1000
2000
0
20
40
60
80
100
cM
Chromosome 8
Number SNPs
0
2000
4000
0
20
40
60
80
100
cM

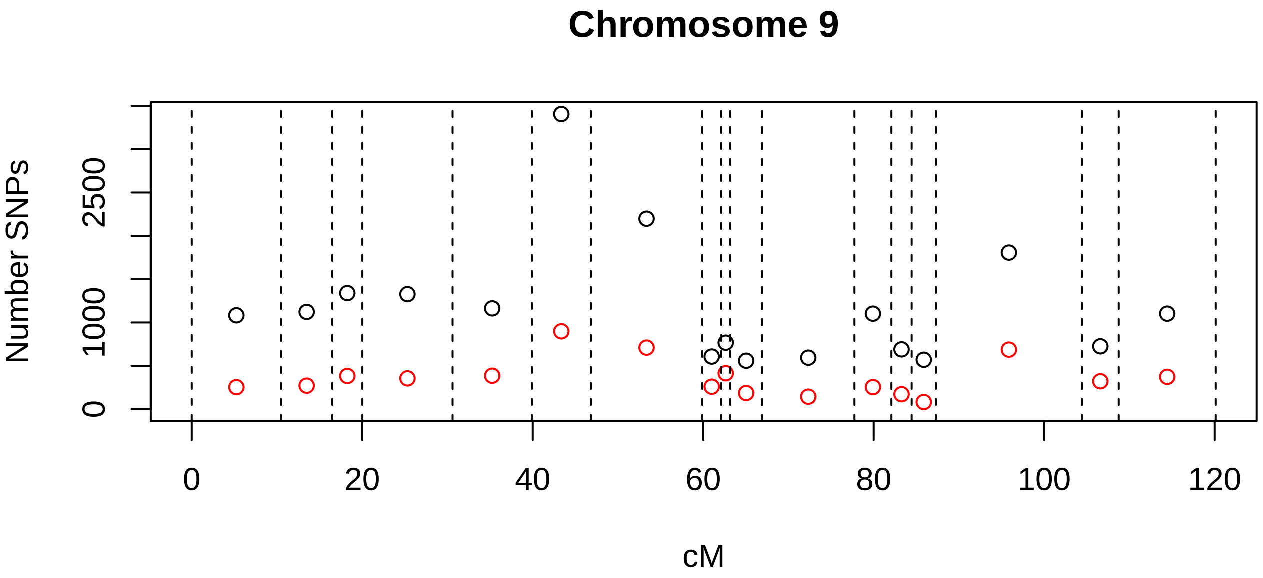
Chromosome 9
Number SNPs
0
1000
2500
0
20
40
60
80
100
120
cM

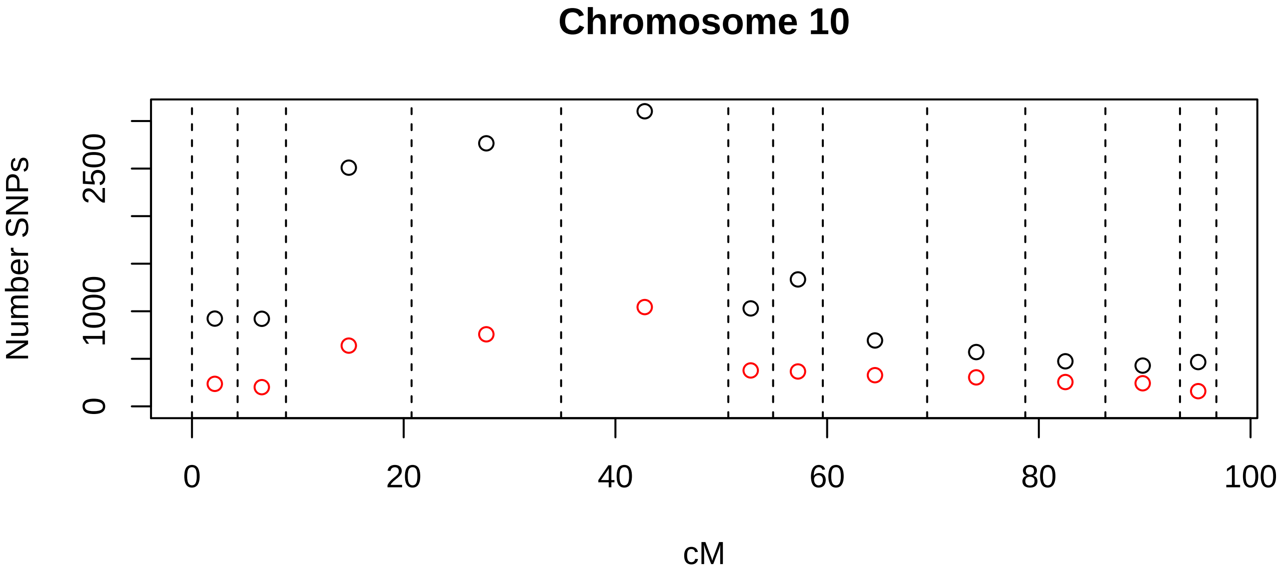
Chromosome 10
Number SNPs
0
1000
2500
0
20
40
60
80
100
cM

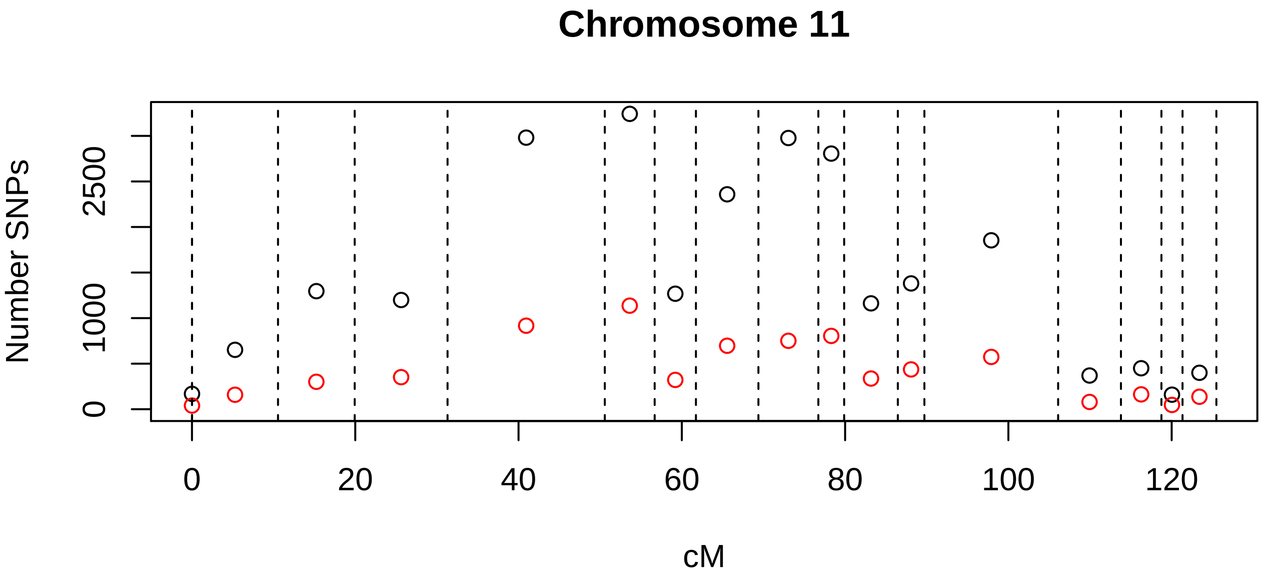

Chromosome 11
Number SNPs
0
1000
2500
0
20
40
60
80
100
120
cM

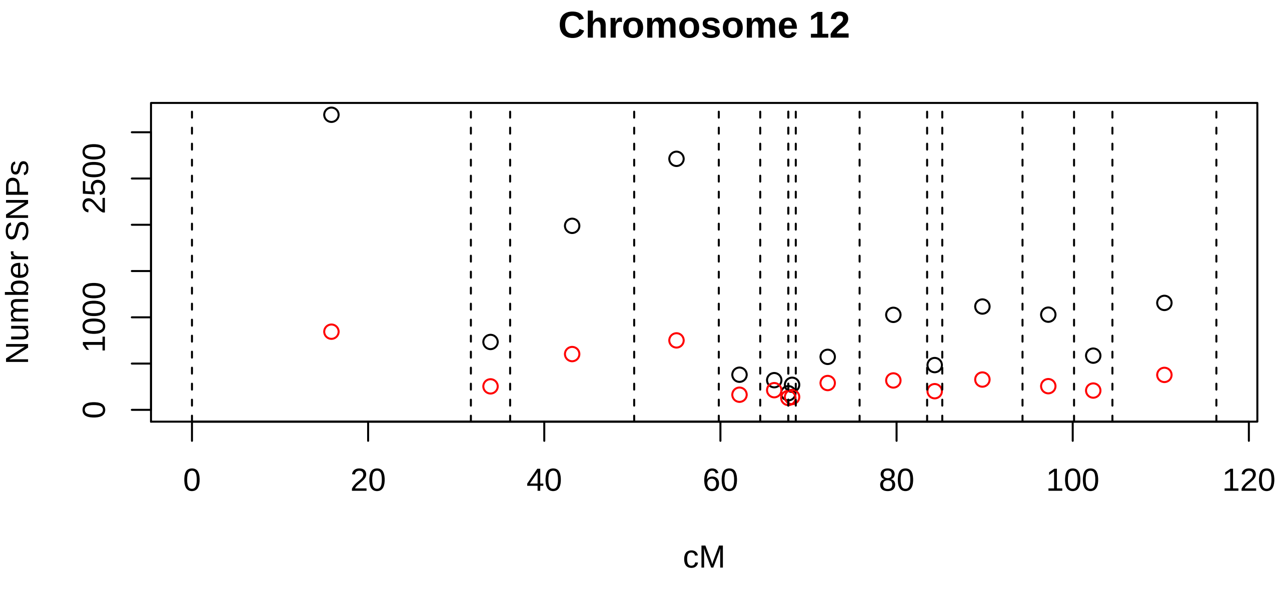

Chromosome 12
Number SNPs
0
1000
2500
0
20
40
60
80
100
120
cM

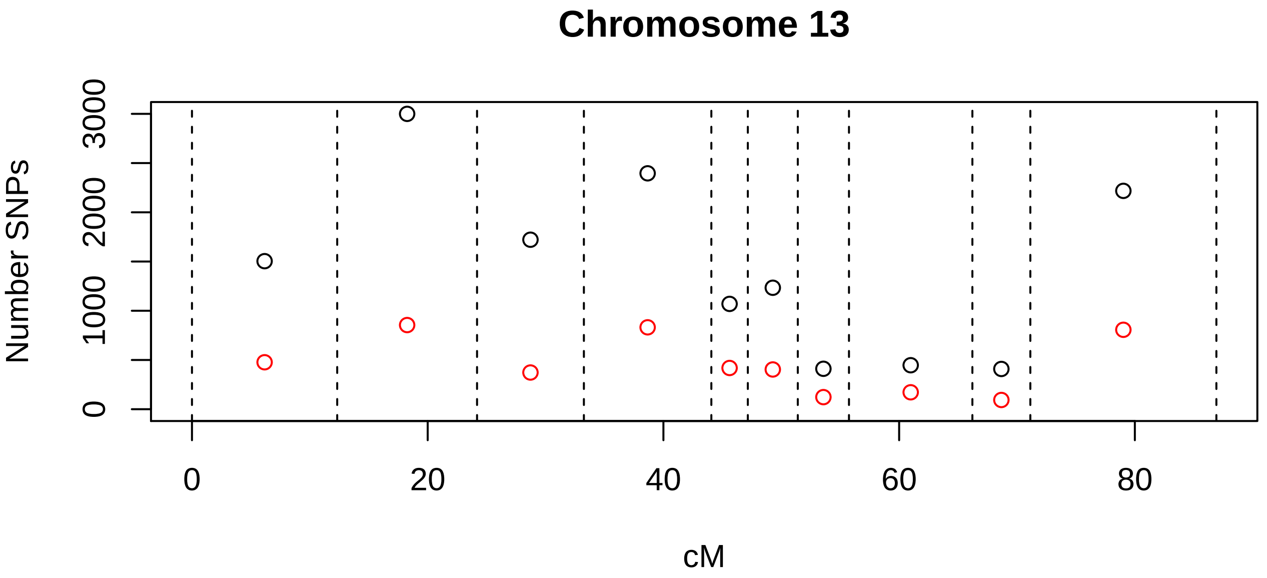
Chromosome 13
Number SNPs
0
1000
2000
3000
0
20
40
60
80
cM

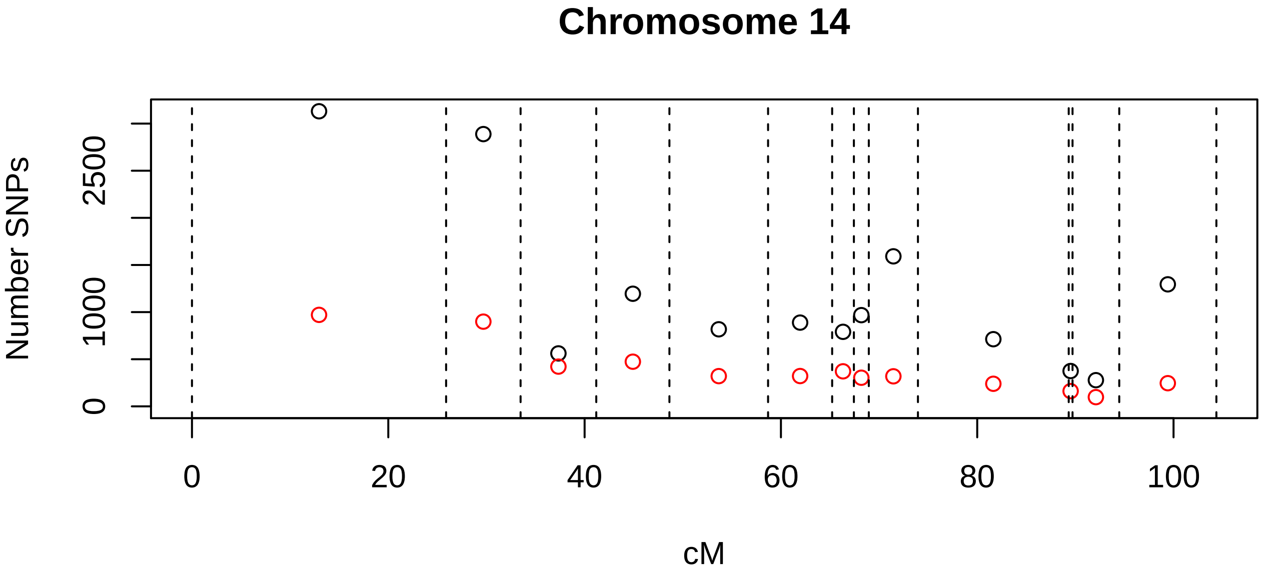
Chromosome 14
Number SNPs
0
1000
2500
0
20
40
60
80
100
cM

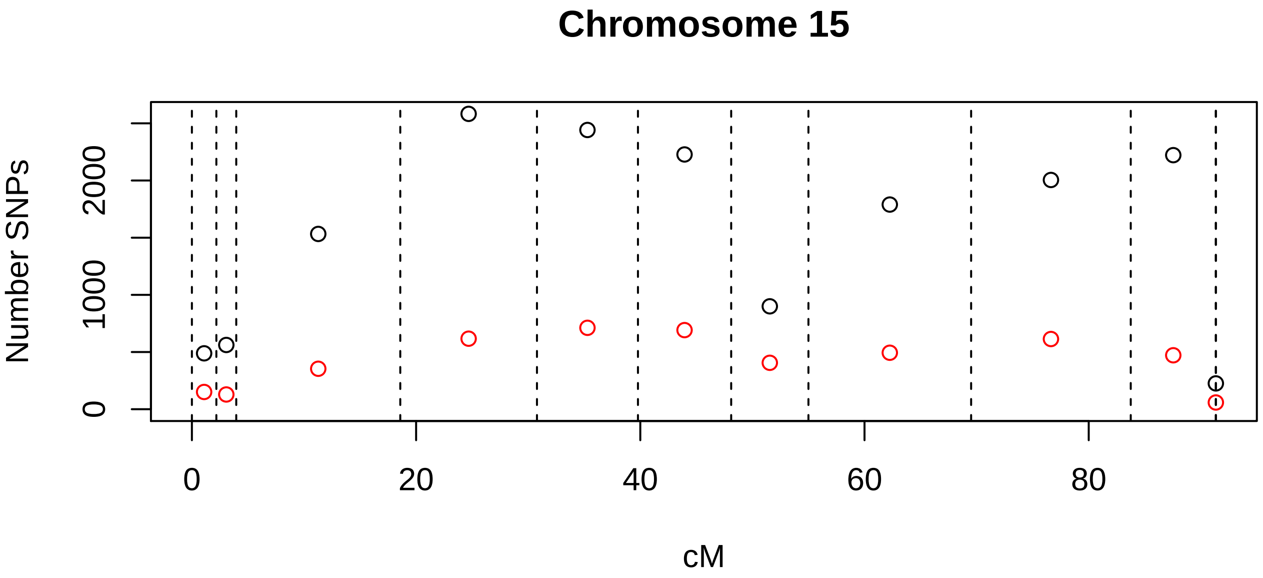
Chromosome 15
Number SNPs
0
1000
2000
0
20
40
60
80
cM

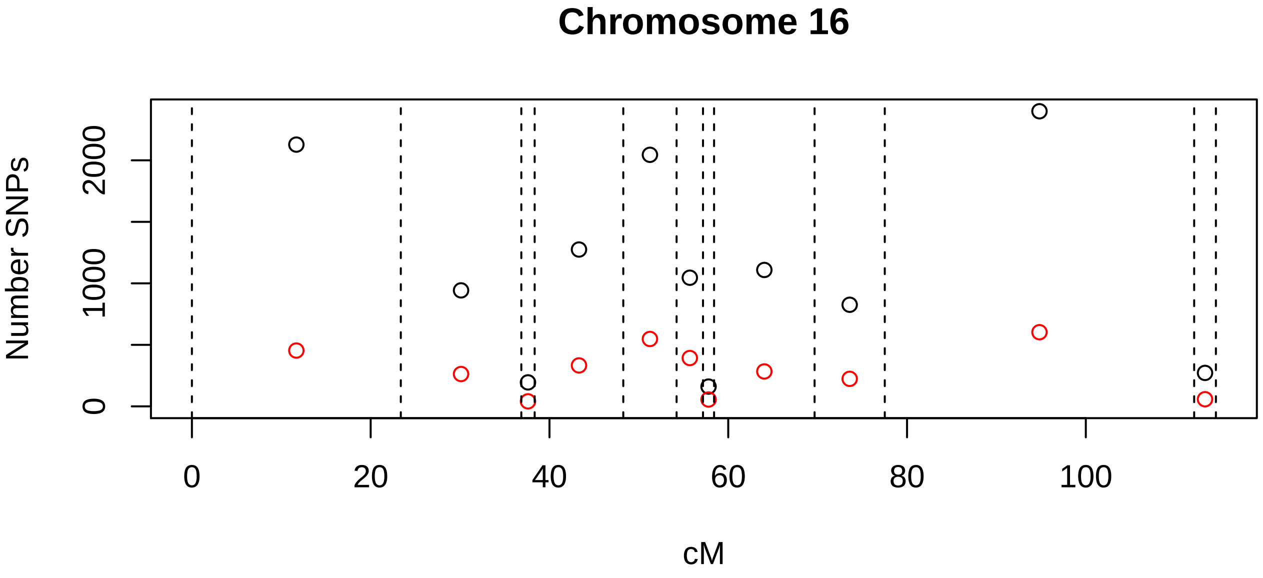
Chromosome 16
Number SNPs
0
1000
2000
0
20
40
60
80
100
cM

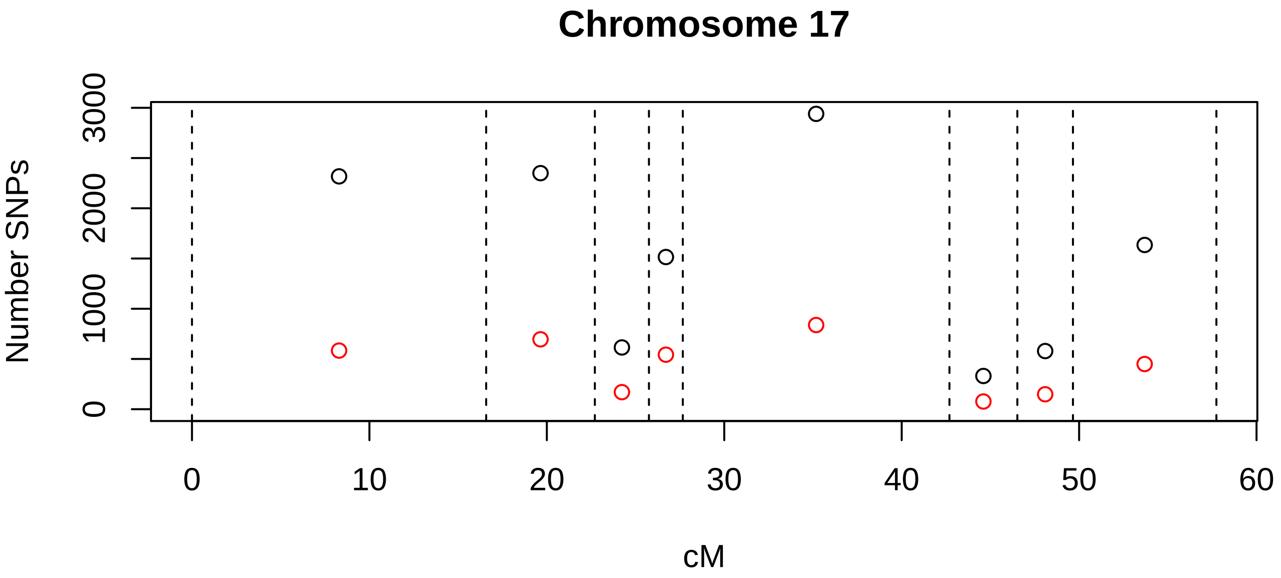
Chromosome 17
Number SNPs
0
1000
2000
3000
0
10
20
30
40
50
60
cM

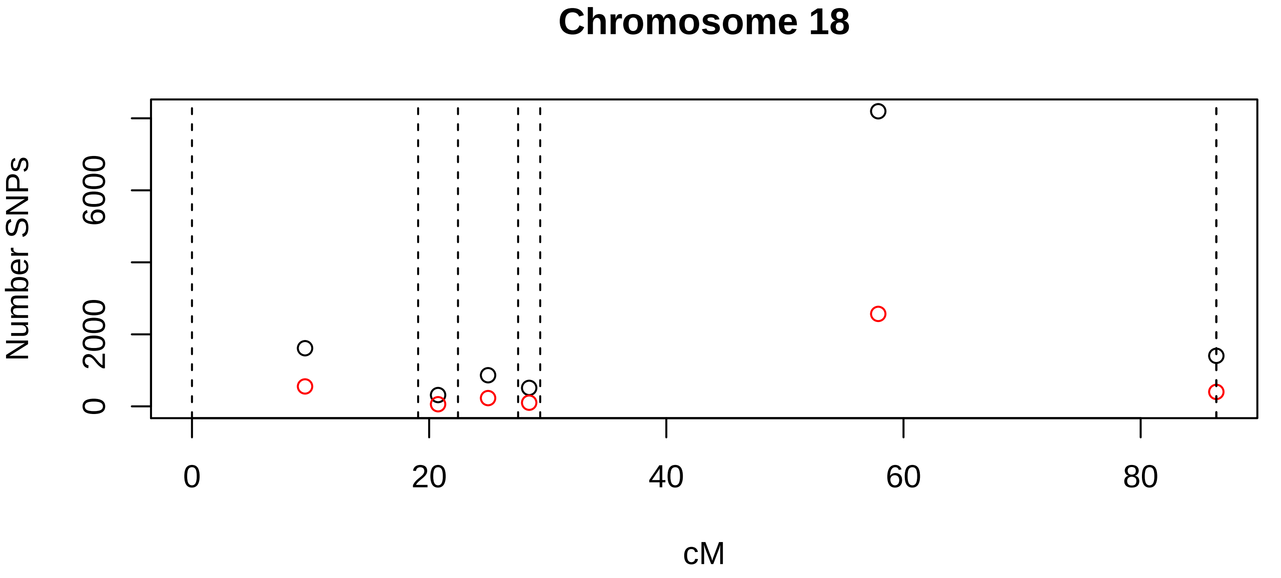
Chromosome 18
Number SNPs
0
2000
6000
0
20
40
60
80
cM

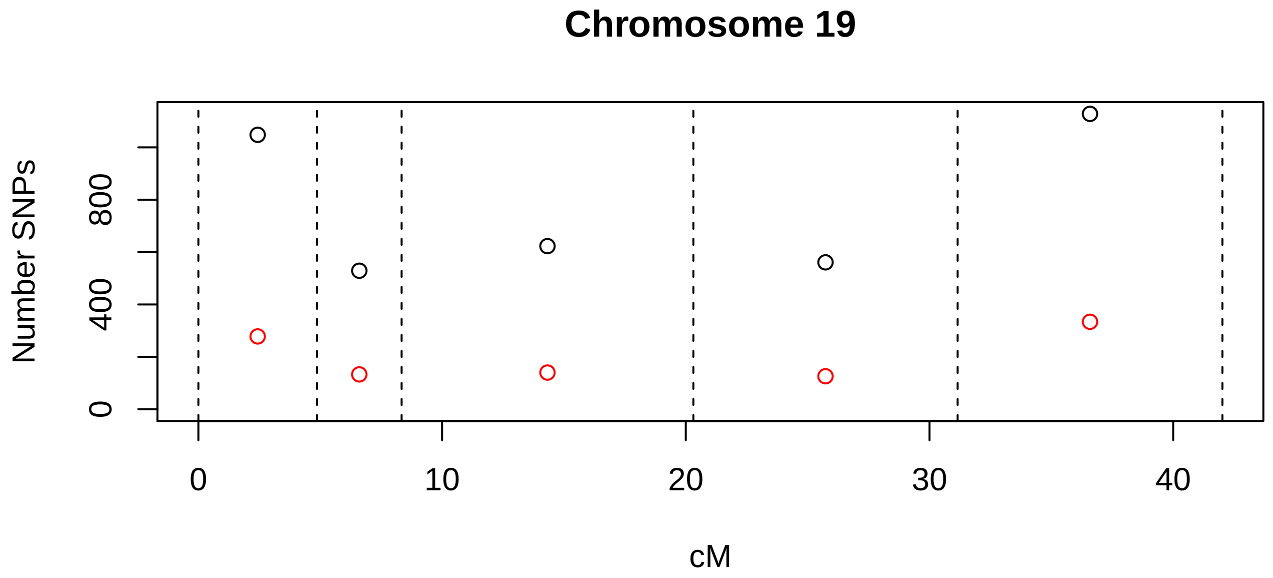
Chromosome 19
Number SNPs
0
400
800
0
10
20
30
40
cM

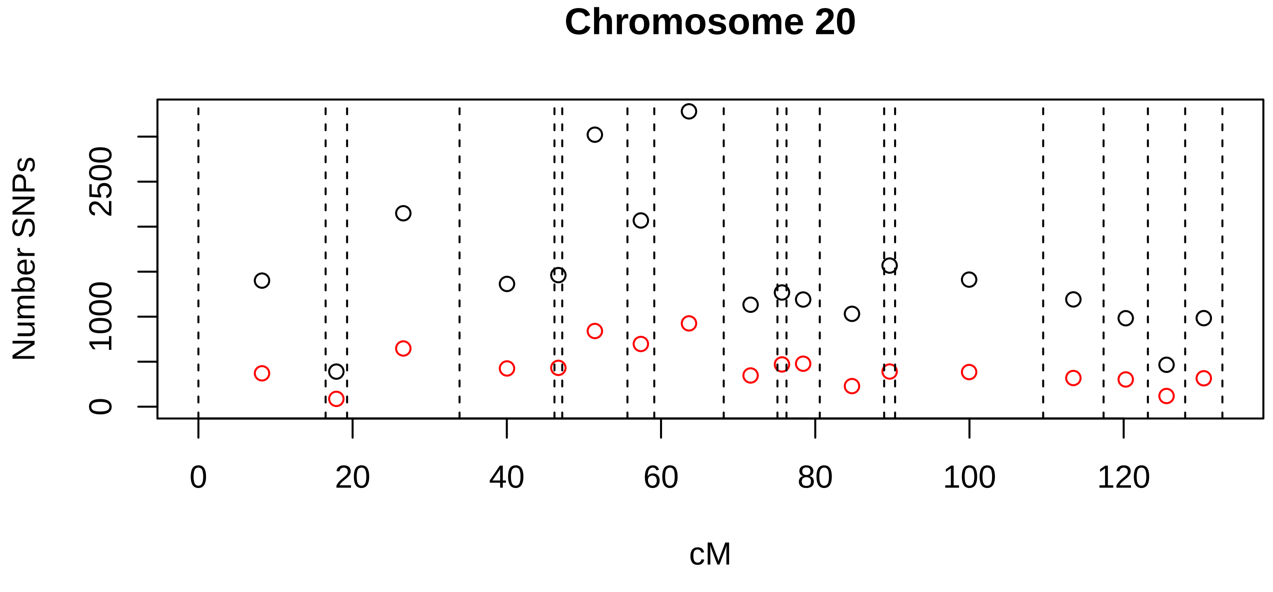
Chromosome 20
Number SNPs
0
1000
2500
0
20
40
60
80
100
120
cM

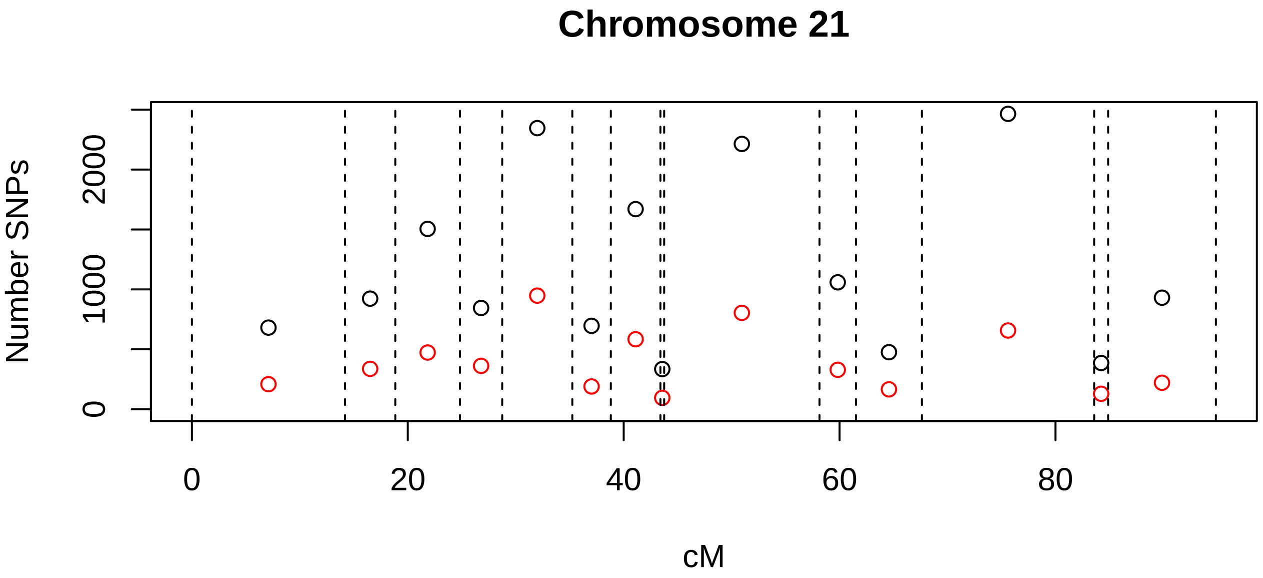
Chromosome 21
Number SNPs
0
1000
2000
0
20
40
60
80
cM

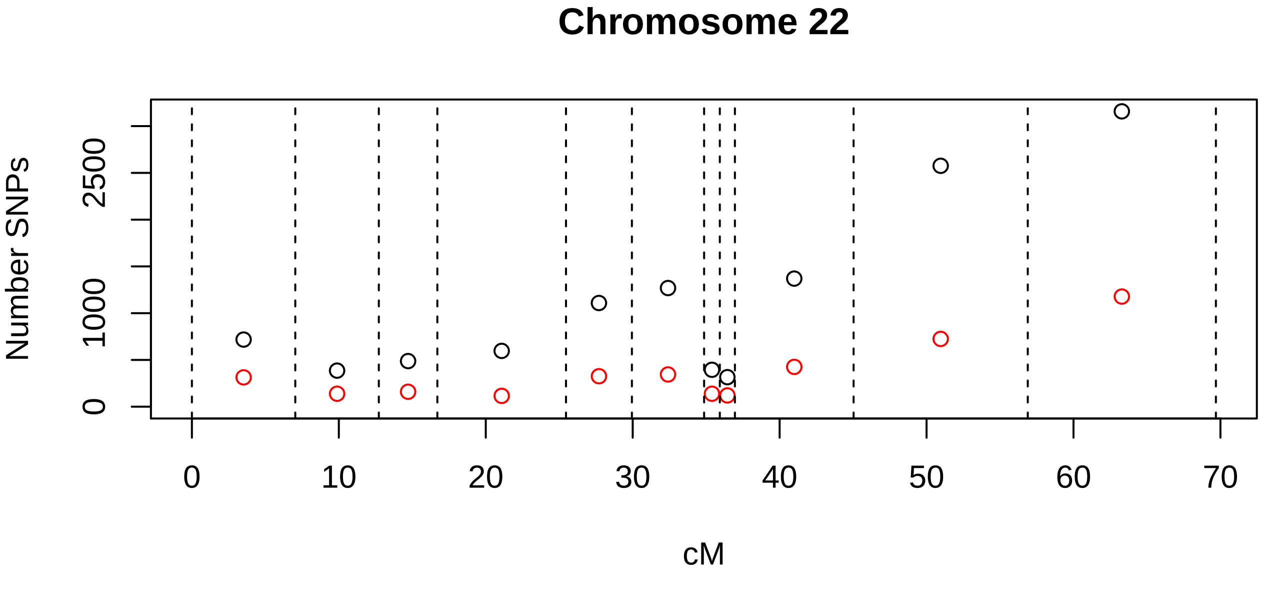
Chromosome 22
Number SNPs
0
1000
2500
0
10
20
30
40
50
60
70
cM

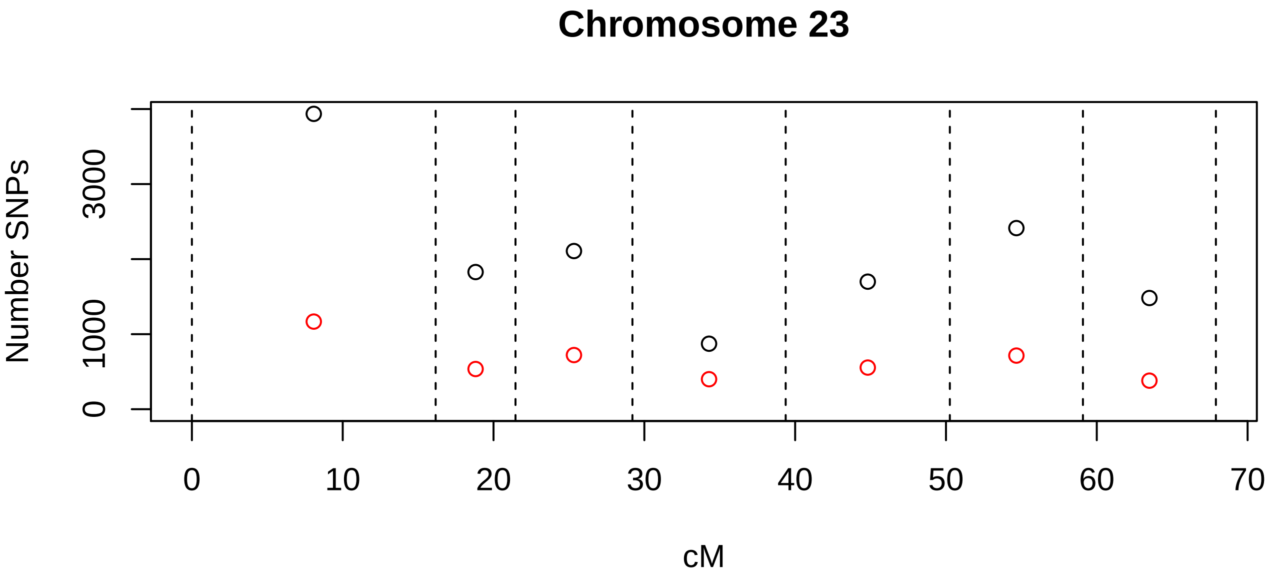

Chromosome 23
Number SNPs
0
1000
3000
0
10
20
30
40
50
60
70
cM

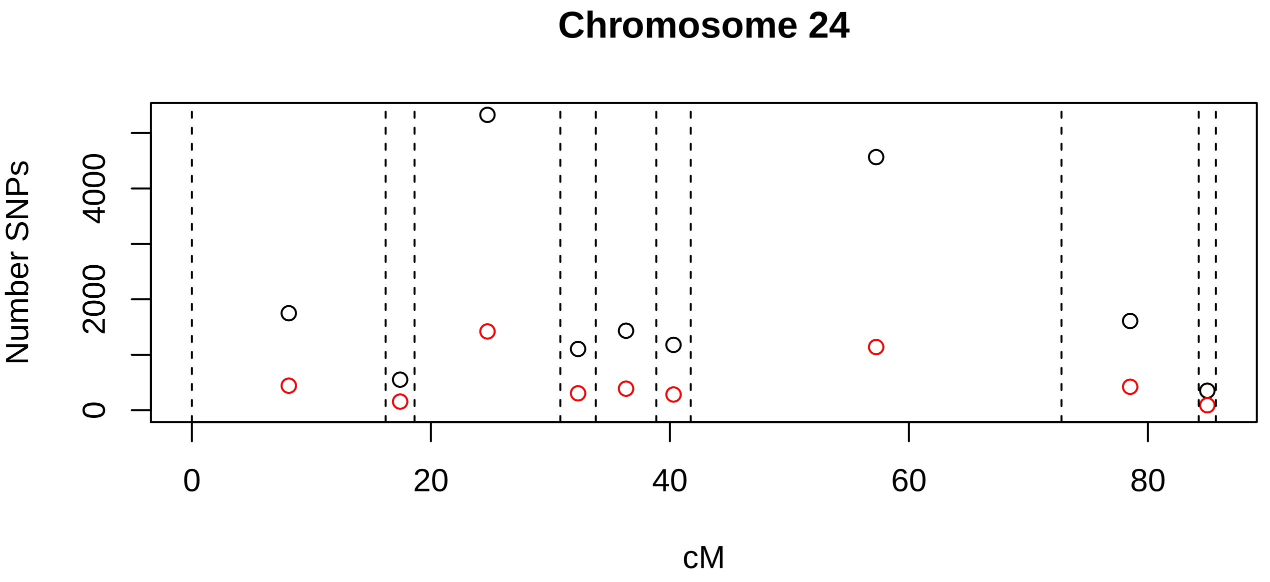

Chromosome 24
Number SNPs
0
2000
4000
0
20
40
60
80
cM

Chromosome 25

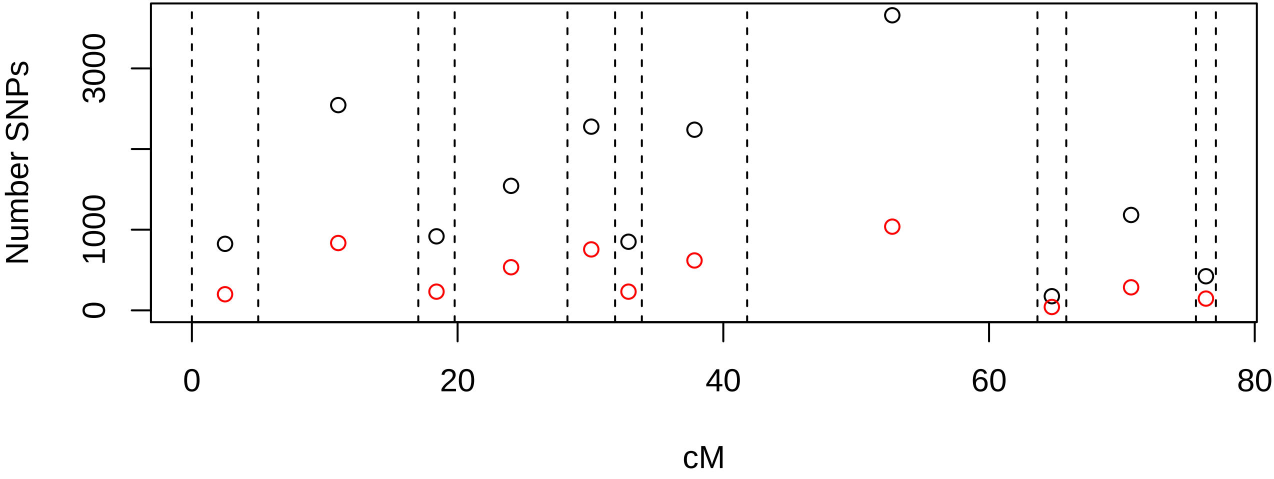

Chromosome 26

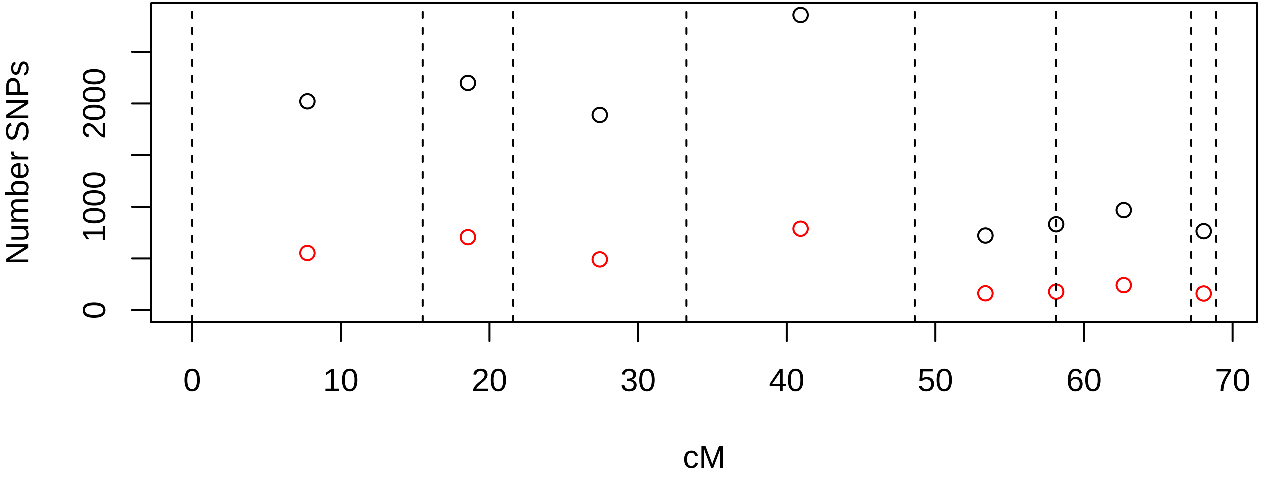

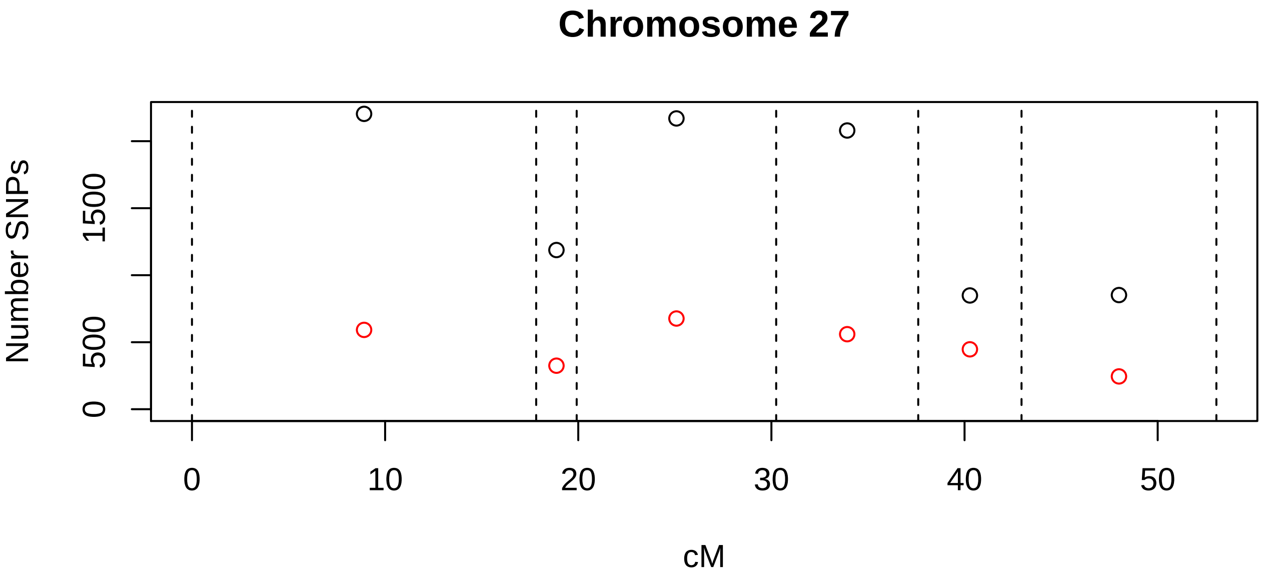
Chromosome 27
Number SNPs
0
500
1500
0
10
20
30
40
50
cM

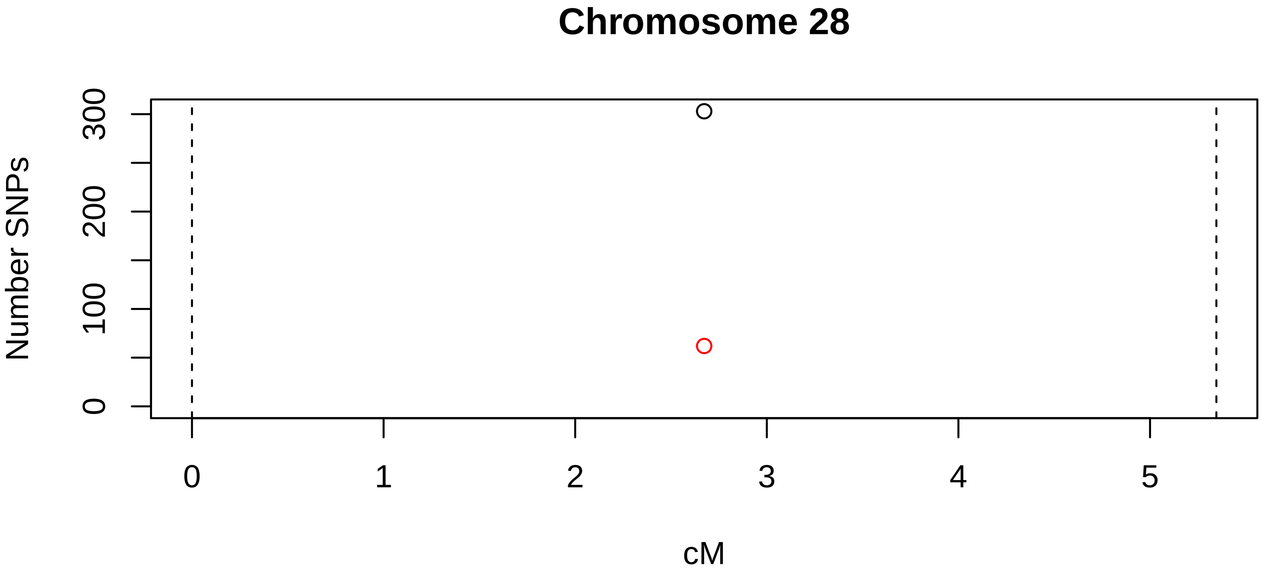
Chromosome 28
Number SNPs
0
100
200
300
0
1
2
3
4
5
cM

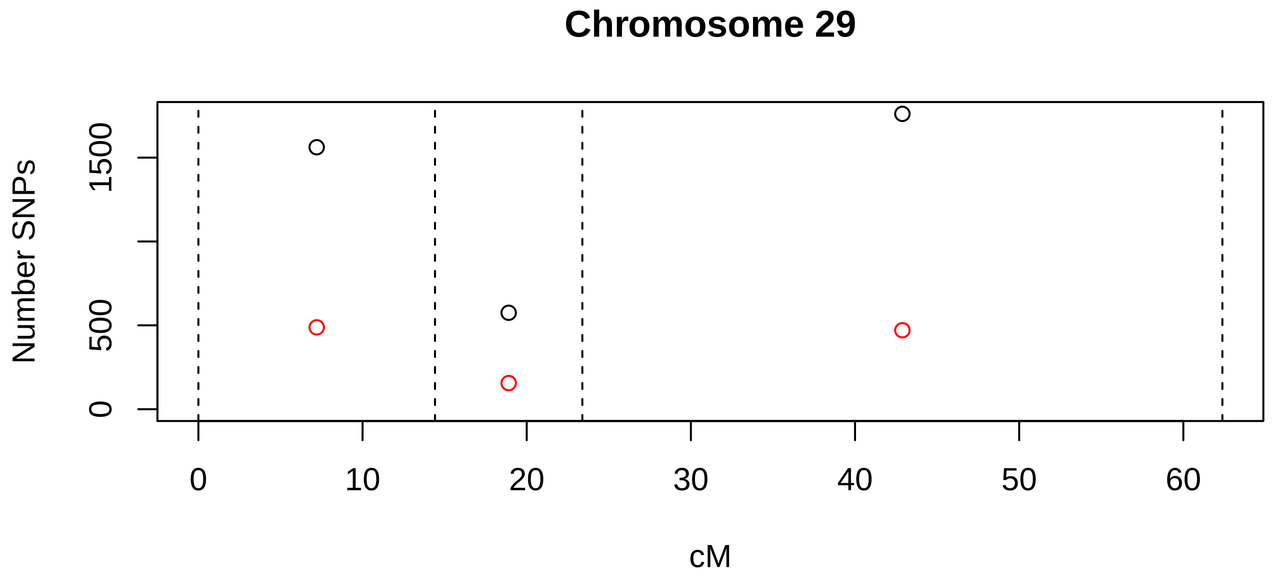

Chromosome 29
Number SNPs
0
500
1500
0
10
20
30
40
50
60
cM
