# Supplementary material for: Sequencing strategies and characterization of 721 vervet monkey genomes for future genetic analyses of medically relevant traits
Source: BMC Biol. 2015 Jun 20;13:41. doi: 10.1186/s12915-015-0152-2 (PMC4494155; doi:10.1186/s12915-015-0152-2)
Supplement: Additional file 3: — Supplementary methods. [file 12915_2015_152_MOESM3_ESM.pdf]

Additional File 3: Supplementary Methods

Read Mapping Protocol – Preliminary Study

The steps in this procedure included read trimming, alignment of all reads to the reference genome via bwa (Li et al. 2009), and marking of duplicates. Read trimming procedures deviated slightly from those of the 1000 Genomes project, as follows: we first smoothed the quality score of one base by replacing it with the median of a running three-base window, trimmed the read head and tail if Phred scores of all bases within the head and tail were below 20, marked the remaining interlacing low-quality (Phred score <=20) bases as missing, and removed reads whose post-trimming length is below 50 or for which more than 30% bases were marked as missing.

Stage 1 filtering steps

We applied the following filters to variant sites identified in Stage 1: base quality Phred score >= 20, mapping quality score >=20, allelic imbalance p-value <= 0.001, strand bias p-value <= 0.001, and variants where the median minimum quality in a window around the variant position falls below 15 will be filtered (Rimmer et al. 2013) for bad reads.

To derive the final set of SNPs we applied additional filters after completing Platypus calling. We discarded loci that (1) were multi-allelic or multi-nucleotide; (2) displayed cumulative coverage outside the 2-fold range of global median coverage; (3) had a minor allele frequency <25%; (4) had any missing calls; (5) were within 5-bp of another SNP.

LiftOver scaffold-based-coordinates to chromosome coordinates

Variants that passed through Stage 2 in the main phase of the project were positioned using scaffold-based coordinates. The lift-over of these variants to chromosome coordinates consisted of the following steps: (1) Extract the flanking sequences of each SNP from the pre-submission reference assembly; (2) Blast/BWA flanking sequences against the NCBI-released reference with no gap allowed, maximum number of mismatches set to two, and no more than one hit; (3) Construct a map between old coordinates and new coordinates based on the alignment result from step 2; (4) Run GATK's LiftOverVariants (DePristo et al. 2011) to change SNP-coordinates to the NCBI-released reference based on the map from step 3. In this step we reverse-complemented the reference and alternative alleles for a SNP if its flanking sequence mapped to the negative strand; (5) Filter LiftOver variants. In this step we discarded SNPs where we detected a mismatch between the reference allele and the base at a particular site (i.e. where there was a base change between the pre-submission and NCBI-released versions of the reference, or if the SNP was inaccurately mapped in the pre-submission version; (6) We removed all SNPs from prior versions of the reference genome that were mapped to the same location in the reference genome version used in the pre-submission version.

Post-LiftOver filters

During the course of checking the validity of variants, we determined that some SNPs reside in poorly-aligned regions due to the presence of repeats, and/or incompleteness of the reference genome. We added additional filters to identify and remove these position-questionable SNPs.

The first new filter relied on a LiftOver mapScore, a statistic quantifying the amount of deviation between one SNP's observed position and its inferred true position. The true position is inferred based on the order and position of neighboring SNPs in the pre-submission reference genome. We used a threshold of mapScore<0.5 to filter loci. We then identified genotype calls that are in regions of poor alignment, defined as either >10% of aligned reads showing mapping quality below 2 or coverage beyond the two-fold (or 10-fold for 1X monkeys) range of global medium depth, and masked all of them as missing. We then removed SNPs that showed >50% missing in monkeys sequenced at 4X and above.

REFERENCES

DePristo et al. 2011 A framework for variation discovery and genotyping using next-generation DNA sequencing data Nat Genet 43:491-98

Li et al. 2009 The sequence alignment/map (SAM) format and SAMtools Bioinformatics 25:2078-9
